# PCL/Chitosan/Bioglass Hybrid Composite Shape Memory Scaffolds With Accelerated Degradation and Enhanced Bioactivity

**DOI:** 10.1002/mame.70250

**Published:** 2026-06-02

**Authors:** Damion T. Dixon, Darian K. Kanu, Melissa A. Grunlan

**Affiliations:** 1 Department of Biomedical Engineering, Texas A&M University, College Station, Texas, USA; 2 Department of Materials Science and Engineering, Texas A&M University, College Station, Texas, USA; 3 Department of Chemistry, Texas A&M University, College Station, Texas, USA

**Keywords:** bioactivity, bioglass, bone regeneration, chitosan, composite scaffold, poly(*ε*-caprolactone), shape memory polymer

## Abstract

“Self-fitting” shape memory polymer (SMP) scaffolds based on cross-linked poly(*ε*-caprolactone)-diacrylate (PCL-DA) represent a promising regenerative strategy for treating irregular craniomaxillofacial bone defects. Beyond conformal fitting, such scaffolds should exhibit intrinsic bioactivity as well as controlled and robust degradation rates. In prior reports, chitosan (CS) and 45S5 Bioglass (BG) were separately incorporated to form SMP scaffolds. “CS-only” hybrid scaffolds suppressed fungal biofilm formation and accelerated degradation, whereas “BG-only” composite scaffolds induced hydroxyapatite (HAp) mineralization and also accelerated degradation. Herein, “hybrid composite” scaffolds incorporating both CS and BG were developed. Semi-interpenetrating networks were fabricated using PCL-DA and CS-*graft*-PCL at 90:10 and 75:25 wt% ratios, corresponding to 1 and 2.5 wt% CS, respectively. BG was introduced at 5 and 10 wt%, and localized on the pore walls. These scaffolds retained excellent shape memory behavior despite a reduction in PCL crystallinity. Degradation was faster versus corresponding BG-only and CS-only scaffolds, and robust HAp mineralization was observed after just 1 day in 1X simulated body fluid. The hybrid composite scaffolds were mechanically robust, and HAp mineralization induced a substantial increase in modulus.

## 1 | Introduction

Biologic and alloplastic grafts used to treat irregular craniomaxillofacial (CMF) bone defects often fail to achieve sufficient bone-to-graft contact, compromising osseointegration and delaying healing [1–3]. A conformally fitting, “off-the-shelf” scaffold that supports bone regeneration offers a promising alternative. Beyond shape conformity, such scaffolds should possess intrinsic functionality to actively guide tissue repair [4]. Key among these are bioactivity to stimulate bone formation and sustained antimicrobial efficacy to mitigate post-operative infection [5, 6]. In addition, scaffolds should support neotissue infiltration by providing adequate porosity, pore interconnectivity, and suitably robust degradation rates [7–9].

We have previously reported biodegradable “self-fitting” (i.e., capable of conformal fitting to irregular geometries) shape memory polymer (SMP) scaffolds composed of poly(*ε*-caprolactone)-diacrylate (PCL-DA, *M*_n_ ~ 10 kg mol^−1^, *T*_m_ or “*T*_trans_” ~55°C) for treating complex CMF bone defects [10–12]. Their fabrication via a fused salt template (i.e., solvent-cast particulate leaching, SCPL) yielded highly porous (~70%) scaffolds with interconnected macropores (~220 μm), conducive to cell and tissue ingrowth. The covalent cross-links serve as netpoints that define the permanent shape, while the crystalline lamellae act as switching segments enabling thermally driven shape actuation. When exposed to warm saline (*T* > *T*_trans_), the crystalline lamellae melt, rendering the scaffold malleable and enabling press-fitting into irregular defects, thereby achieving reversible conformal fitting via shape memory behavior. Shape recovery (*R*_*r*_) then drives expansion of the scaffold to the defect perimeter, ensuring high bone-to-scaffold contact (i.e., providing a conformal fit). Cooling to body temperature (*T* < *T*_trans_) restores rigidity as the lamellae recrystallize, locking the scaffold into its new shape through shape fixity (*R*_*f*_). Although conformally fitting, these PCL-based SMP scaffolds lack prerequisite bioactive and antimicrobial attributes, which may limit their capacity to promote bone growth and reduce infection risk [13, 14].

To address these limitations, we have developed complementary strategies to enhance scaffold functional performance: first, through bioactive composites with 45S5 Bioglass (BG) [15], a widely used material with potent osteogenic and bioactive properties [16–18], and more recently through “hybrid” scaffolds incorporating chitosan (CS) [19], a natural biopolymer with inherent antimicrobial behavior [20]. BG-based composite SMP scaffolds were fabricated using a modified SCPL method with a fused salt/BG template. This approach led to the concentration of BG on the pore walls, rather than embedded within the struts. In this way, low BG loadings (5 and 10 wt%) were sufficient to achieve rapid bioactivity, as evidenced by hydroxyapatite (HAp) mineralization within 1 day in 1X simulated body fluid (SBF), while avoiding brittleness. Additionally, degradation rates increased with BG loading. In our more recent strategy, hybrid CS/PCL SMP scaffolds were formed as semi-interpenetrating networks (semi-IPNs) by combining cross-linked PCL-DA and thermoplastic (i.e., non-cross-linked) CS-*graft*-PCL (1:6, 1:12, 1:24, or 1:48 molar ratio), each at 90:10 and 75:25 wt% [19]. This resulted in eight CS/PCL scaffolds with varying CS content (~0.8 to ~7 wt%). Scaffolds based on CS-*graft*-PCL (1:24) exhibited the fastest degradation, likely due to intermediate phase separation facilitating greater water uptake [21]. While the CS/PCL scaffolds with CS contents as low as ~0.8 wt% sufficiently suppressed fungal colonization, they were not bioactive. Thus, “hybrid composite” SMP scaffolds that include both BG and CS could synergistically provide bioactivity, antimicrobial behavior, and accelerated degradation.

**FIGURE 1 |:**
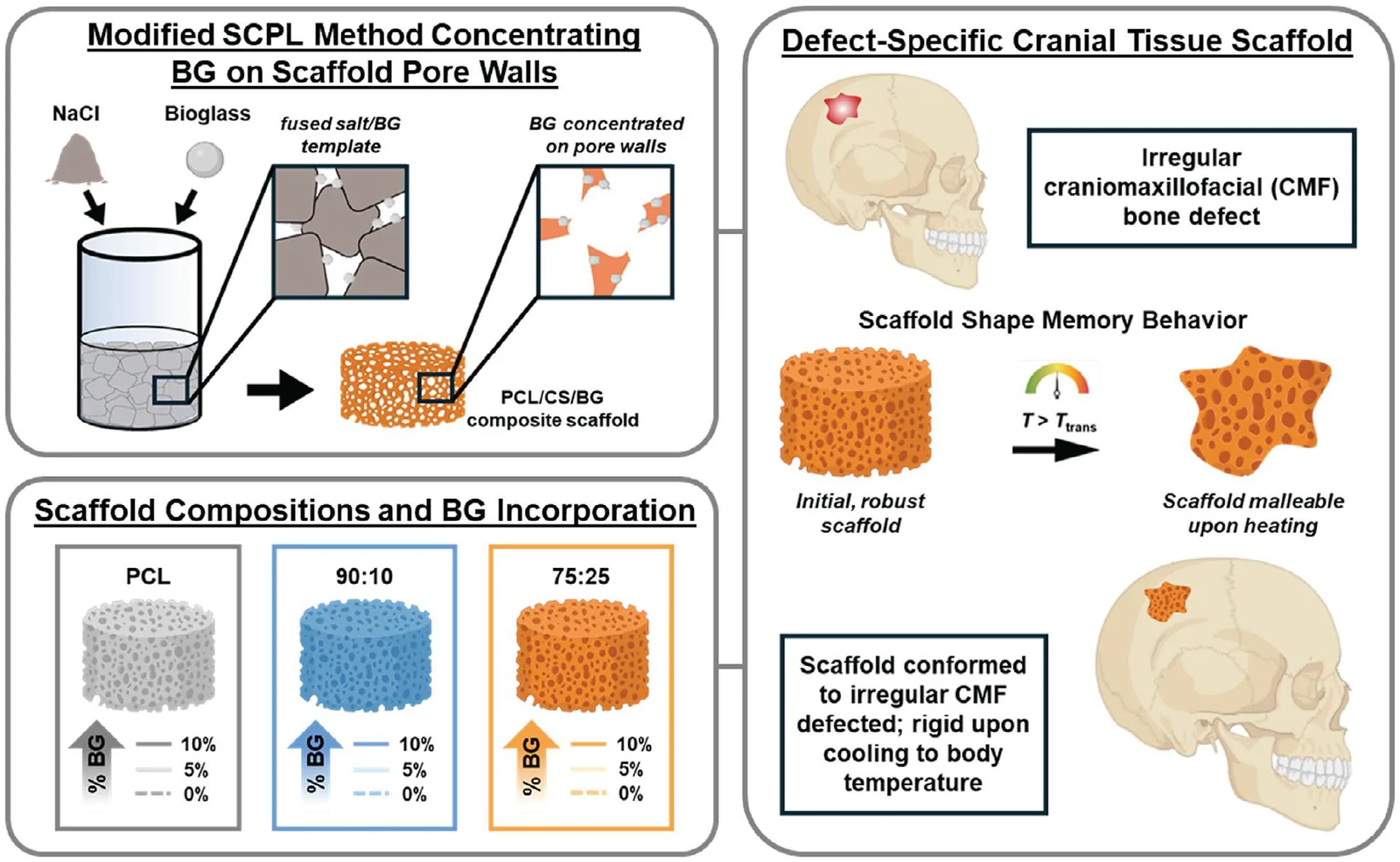
Hybrid composite SMP scaffolds containing both BG and CS (90:10%–5%, 90:10%–10%, 75:25%–5%, and 75:25%–10%) were fabricated and compared to PCL-only (PCL-0%), BG-only (PCL-5% and PCL-10%), and CS-only (90:10%–0% and 75:25%–0%) SMP scaffolds. Hybrid composite scaffolds and analogous non-composites were formed as semi-IPNs from PCL-DA/CS-*graft*-PCL (1:24 molar ratio CS:PCL for graft copolymer) at either 90:10 or 75:25 wt%.

Herein, to further accelerate degradation behavior and to impart bioactivity, we report the formation of hybrid composite PCL/CS/BG SMP scaffolds (Figure 1; scaffold compositions and notations are summarized in Table S1). Scaffolds were prepared using CS-*graft*-PCL (1:24 molar ratio) [19], selected for its robust degradation, partly attributable to its partial miscibility with PCL [21]. PCL-DA and CS-*graft*-PCL (1:24) were combined at 100:0, 90:10, and 75:25 wt% (coinciding to 0, 1, and 2.5 wt% CS, respectively), each with BG incorporated at 0, 5, and 10 wt%. To isolate the individual and synergistic contributions of BG and CS, composite scaffolds were compared to analogous formulations without BG, and CS-containing hybrids were further evaluated against PCL controls. Scaffold morphological, thermal, shape memory, and compressive mechanical properties were characterized, along with in vitro degradation and bioactivity (i.e., in vitro HAp mineralization). The impact of scaffold mineralization on mechanical properties was also assessed.

## 2 | Materials and Methods

### 2.1 | Materials

CS [purified powder, *M*_w_ = 15 kg mol^−1^ per manufacturer, % degree of deacetylation (DDA) = 86%, calculated from ^1^H-NMR spectroscopy] was purchased from Polysciences, Inc. (Warrington, PA, USA) and vacuum-dried (50°C, 30 in. Hg) for 24 h prior to use. Methanesulfonic acid (MeSO_3_H), *ε*-caprolactone, potassium phosphate monobasic (KH_2_PO_4_), sodium hydroxide (NaOH), poly(*ε*-caprolactone) diol (PCL-diol, *M*_n_ ~ 10 kg mol^−1^ per manufacturer), reagent-grade dichloro methane (DCM), 4-(dimethylamino)-pyridine (DMAP), triethylamine (Et_3_N), acryloyl chloride, ethyl acetate, potassium carbonate (K_2_CO_3_), anhydrous magnesium sulfate (MgSO_4_), deuterated chloroform (CDCl_3_), sodium chloride (NaCl), 2,2-dimethoxy-2-phenylacetophenone (DMP), 1-vinyl-2-pyrro lidinone (NVP), reagent alcohol (ethanol), sodium bicarbonate (NaHCO_3_), potassium chloride (KCl), potassium phosphate dibasic trihydrate (K_2_HPO_4_·3H_2_O), magnesium chloride hexahydrate (MgCl_2_·6H_2_O), hydrochloric acid (HCl), calcium chloride (CaCl_2_), sodium sulfate (Na_2_SO_4_), and tris(hydroxymethyl)-aminomethane (tris) were purchased from Sigma-Aldrich (St. Louis, MO, USA). BG (10 μm) was purchased from Mo-Sci Corp (Rolla, MO, USA). Deionized (DI) water (18 MΩ) was acquired from an in-house purification system. Prior to use, all solvents were dried over 4 Å molecular sieves (Sigma-Aldrich), and all reagents were vacuum-dried [room temperature (RT), overnight (ON), 30 in. Hg].

### 2.2 | Synthesis

Reactions were run under positive nitrogen (N_2_) pressure utilizing oven-dried (120°C, ON) glassware and Teflon-covered magnetic stir bars. After synthesis and subsequent purification, polymer structures (including the *M*_n_ and % acrylation of PCL-DA) were confirmed with ^1^H-NMR spectroscopy (Avance NEO 400 MHz spectrometer) operating in the Fourier Transform mode with CDCl_3_ as the standard.

CS-*graft*-PCL was synthesized via ring-opening polymerization as previously reported [19]. Briefly, 1.0 g of thoroughly dried CS (6.0 mmol of glucosamine units, accounting for the % DDA) and 15 mL of MeSO_3_H were combined in a 100 mL round-bottom (rb) flask equipped with a rubber septum and allowed to stir at 45°C until the CS had completely dissolved. After purging with N_2_ for ~3 min, *ε*-caprolactone (16.4 g, 143.7 mmol, 24 equiv) was injected into the flask. The reaction was allowed to proceed at 45°C under positive N_2_ pressure to remove moisture and prevent homopolymerization. After ~5 h, the reaction mixture was precipitated into an ice-cold buffer solution (100 mL of 0.2 M KH_2_PO_4_, 16 mL of 10 M NaOH, and 100 g of crushed ice) to neutralize the MeSO_3_H. The precipitate was collected by vacuum filtration and washed several times with DI water to remove the remaining salts. After vacuum drying (RT, ON, 30 in. Hg), the isolated CS-*graft*-PCL was characterized similarly and was found to be in good agreement with our prior report (>85% yield, ~10% CS, *M*_*n*_ ~ 14 kg mol^−1^, and *M*_*w*_ ~ 31 kg mol^−1^) [19]. The graft copolymer was stored in a vacuum desiccator until use.

PCL-DA was prepared by acrylation of PCL-diol (*M*_n_ ~ 10 kg mol^−1^) similar to that previously reported [11]. Briefly, 20.0 g of PCL-diol and 120 mL of DCM were combined in a 250 mL rb flask equipped with a rubber septum and allowed to stir at RT until the PCL-diol had completely dissolved. DMAP (6.6 mg) was then added as a catalyst and allowed to fully dissolve. After purging the flask with N_2_ for ~3 min, Et_3_N (0.56 mL, 4.0 mmol) and acryloyl chloride (0.65 mL, 8.0 mmol) were added dropwise in succession. The reaction was allowed to proceed at RT under positive N_2_ pressure for 30 min prior to being refluxed at 55°C for ~20 h. The reaction was quenched with removal from heat, and the DCM was removed using a rotary evaporator. The crude product was then dissolved in 135 mL of ethyl acetate and gravity filtered. After filtering, the ethyl acetate was removed by rotary evaporation. The isolated PCL-DA was then dissolved in 135 mL of DCM, washed with 13.5 mL of 2 M K_2_CO_3_, and allowed to separate ON in a separatory funnel. After separation, the organic layer was collected, dried with MgSO_4_, gravity filtered, dried using a rotary evaporator, and finally vacuum-dried (RT, ON, 30 in. Hg). ^1^H-NMR of the purified product (~80% yield) was consistent with that reported previously (*M*_*n*_ ~ 10 kg mol^−1^ and >90% acrylation) [22].

### 2.3 | Fabrication

#### 2.3.1 | Scaffolds

Porous scaffolds were fabricated via an established SCPL procedure employing a fused salt/BG template (a.k.a. “glass-in-salt mold”) to form composite scaffolds with BG concentrated on the pore walls [15]. Briefly, sieved NaCl particles (10 g, 460 ± 60 μm) and the specified amount of BG (0, 5, or 10 wt%) were added into 20 mL scintillation vials and thoroughly mixed. Then, in four equal portions, DI water (0.81 mL) was added and mixed vigorously with a glass rod. After compaction of the NaCl and BG, vials were capped and subjected to centrifugation (3220 rpm, 15 min) to create the fused templates. Uncapped vials were then dried under vacuum (RT, ON, 30 in. Hg) to remove the DI water.

Macromer solutions (i.e., precursor solutions prior to cross-linking) were prepared in DCM (0.15 g mL^−1^) and a photoinitiator (10 wt% DMP in NVP) was added at 15 vol% and mixed on a shaker plate (150 rpm, ~3 min). Approximately 5 mL of each macromer solution was added to individual fused templates prior to the vials being capped and centrifuged (1260 rpm, 10 min) to facilitate dissolution throughout. Then, to cross-link the PCL-DA, the vials were exposed to UV light (UV-Transilluminator, 6 mW cm^−2^, 365 nm) for ~6 min. Uncapped vials were once again dried under vacuum (RT, ON, 30 in. Hg) to remove the DCM prior to NaCl leaching. This was achieved by soaking in water and ethanol (1:1 by vol) for 5 days with daily solution changes. The resulting porous scaffolds were air-dried (RT, ~4 h), vacuum-dried (RT, ON, 30 in. Hg), and annealed under vacuum (85°C, 1 h, 30 in. Hg). Shrunken scaffolds (*d* ~ 12 mm) were sliced into discs (*t* ~ 2 mm) using a vibratome (Leica Biosystems VT 1000 S) and subsequently biopsy-punched (Integra Miltex, 6 mm). The final dimensions of each scaffold specimen was *d* ~ 6 mm × *t* ~ 2 mm.

#### 2.3.2 | Films

Analogous solid films were prepared from the aforementioned macromer solutions for porosity calculations (with and without BG). Macromer solutions with BG were stirred (500 rpm, RT, ON) in a rb flask to allow dispersion. The photoinitiator (15 vol%) was added to the macromer solutions and thoroughly mixed. Approximately 2.5 mL of each solution was then transferred to into circular silicone molds (McMaster-Carr, *d* ~ 45 mm × *t* ~ 2 mm) secured between two glass slides. Molds were then exposed to UV-light for ~3 min on each side to allow the PCL-DA to cross-link. Cured films were carefully removed from molds and air-dried (RT, ON) inside of a fume hood. Films were then soaked in an ethanol bath atop a shaker plate (150 rpm, ~4 h) before being air-dried (RT, ~2 h), vacuum-dried (RT, ~4 h, 30 in. Hg), and annealed under vacuum (85°C, 1 h, 30 in. Hg). A biopsy punch was similarly used, yielding film specimens with final dimensions of *d* ~ 6 mm × *t* ~ 2 mm.

### 2.4 | Sol Content

Scaffolds (*n* = 3) were individually submerged in 10 mL of DCM in sealed 20 mL scintillation vials placed atop a shaker plate (48 h, 150 rpm). Scaffolds were removed from vials, air-dried (RT, ON) in a fume hood, and finally dried under vacuum (RT, ON, 30 in. Hg). Scaffold initial and final masses were used to determine sol content (i.e., % mass loss attributed to the non-cross-linked fraction of the scaffold).

### 2.5 | Thermogravimetric Analysis (TGA)

TGA (Q50, TA Instruments) of scaffold specimens (*n* = 3, *d* ~ 4 mm × *t* ~ 2 mm) was performed from RT to 600°C in platinum pans under N_2_, at a heating rate of 10°C min^−1^.

### 2.6 | Morphology: Pore Size, Porosity (%), and Pore Interconnectivity (%)

Scaffold pore morphology was evaluated via scanning electron microscopy (SEM, Tescan Vega 3, 10 kV accelerating voltage). Prior to imaging, scaffold cross sections were coated with Pt-Pd (~10 nm) using a Cressington 208HR sputter coater. Based on SEM images (*n* = 10), the average pore size was determined from measurements of pores along the diagonal midline utilizing ImageJ software.

After calculating the densities of scaffolds and films for each composition gravimetrically, the % porosity of scaffolds (*n* = 3) was determined using Equation (1):

(1) $$\text{Porosity}\;\left(\%\right)=\frac{\rho_{\text{solid}\;\text{film}}-\rho_{\text{porous}\;\text{scaffold}}}{\rho_{\text{solid}\;\text{film}}}\times 100$$

Pore interconnectivity was calculated using an established water wicking test [23]. Briefly, scaffolds (*n* = 3) were individually submerged in 10 mL of DI water in sealed 20 mL scintillation vials atop a shaker plate (24 h, 150 rpm) to remove air from the pores. Once removed from vials, scaffolds were weighed (mass_total_). After using a Kimwipe to wick away interconnected water, scaffolds were weighed once again (mass_interconnected_). The % pore interconnectivity of scaffolds was calculated using Equation (2):

(2) $$\text{Pore}\;\text{Interconnectivity}\;\left(\%\right)=\frac{{\text{mass}}_{\text{total}}-{\text{mass}}_{\text{interconnected}}}{{\text{mass}}_{\text{total}}}\times 100$$

### 2.7 | Thermal Properties

PCL *T*_m_ and % crystallinity within scaffolds (*n* = 3) was determined using differential scanning calorimetry (DSC, Q250, TA Instruments). Scaffold portions (~10 mg) were hermetically sealed before being heated/cooled (5°C min^−1^, from 0°C to 80°C) over two cycles. To eliminate thermal history, reported values were obtained using the second heating cycle. The *T*_m_ was determined from the endothermic melt peak’s maximum value, while % crystallinity (% X_c_) was quantified using Equation (3):

(3) $$\%\;X_{c}=\frac{{\Delta H}_{\text{m}}}{\Delta {\text{H}}_{\text{c}}^{{}^{\circ}}\times w}\times 100$$

where Δ*H*_m_ is the enthalpy of fusion calculated from the integral of the endothermic melt peak, Δ*H*°_c_ is the enthalpy of fusion for 100% crystalline PCL (139.5 J g^−1^) [24], and *w* is the mass fraction of the scaffold that is PCL (per Table S1).

### 2.8 | Shape Memory: “Self-Fitting” Behavior

The “self-fitting” behavior of scaffolds (*n* = 3) was quantitatively assessed by mimicking implantation into a model defect (UHMWPE, McMaster-Carr, *d* = 5 mm × *t* = 2 mm). Scaffold initial diameters were measured prior to submersion (~1 min) in a preheated (*T* = 60°C) water bath. Next, the malleable scaffolds were press-fit into the model defect. Once cooled to RT (~2 min), the scaffolds were removed from the mold and their diameters recorded to assess shape fixation. Thereafter, scaffolds were resubmerged (~1 min) in the heated bath to allow for shape recovery. After a brief waiting period (~2 min), scaffold diameters were recorded to assess shape recovery. This process was completed again to remove any thermal history of the scaffold. The % shape fixity (*R*_*f*_) and % shape recovery (*R*_*r*_) of scaffolds were obtained for the second cycle (*N* = 2) using Equations (4) and (5), respectively:

(4) $$R_{f}\left(N\right)=\frac{\varepsilon_{u}\left(N\right)}{\varepsilon_{m}}\times 100$$

(5) $$R_{r}\left(N\right)=\frac{\varepsilon_{i}\left(N\right)}{\varepsilon_{r}\left(N\right)}\times 100$$

where *ε*_*u*_(*N*) is the scaffold diameter after being press-fit into the mold, *ε*_*m*_ is the diameter of the mold, *ε*_*i*_(*N*) is the diameter of the scaffold after shape-recovering, and *ε*_*r*_(*N*) is the initial diameter of the scaffold. Diameters were measured via an electronic caliper with a resolution of 0.01 mm.

### 2.9 | Degradation

Degradation testing was performed under base-catalyzed conditions (0.2 m NaOH) according to an adapted ASTM F1635 protocol, using alkaline conditions to accelerate hydrolytic degradation for comparative analysis. Scaffolds (*n* = 3 per time point) were individually submerged in 10 mL of NaOH in sealed 20 mL scintillation vials stored in a 37°C incubator (VWR Benchtop Shaking Incubator, Model 1570, 60 rpm). At daily time points for 7 days, scaffolds were removed, gently rinsed with DI water, and vacuum-dried (RT, ON, 30 in. Hg). The final dry masses were compared to the initial dry masses to determine % mass loss. Scaffolds were not used for subsequent time points.

### 2.10 | Bioactivity

Bioactivity testing for HAp mineralization was conducted in 1X SBF prepared according to Kokubu [25]. Scaffolds (*n* = 5 per time point) were individually submerged in 10 mL of SBF in sealed 50 mL (conical) centrifuge tubes stored in a 37°C water bath for either 1 day or 2 weeks. After removal from the centrifuge tubes, scaffolds were rinsed with DI water and dried under vacuum (RT, ON, 30 in. Hg). Prior to SEM imaging, scaffold cross sections were coated with Pt-Pd (~10 nm). Energy-dispersive X-ray spectroscopy (EDS, Oxford Instruments, 10 kV) was used to analyze calcium-to-phosphorus (Ca/P) molar ratios of HAp mineralization on individual scaffold pore wall surfaces.

### 2.11 | Compression Mechanical Testing

To determine compressive mechanical properties, scaffolds (*n* = 5) were subjected to static compression testing (Instron 5944) at RT up to 85% strain. Each scaffold was pre-loaded with ~0.1 N before applying a constant strain rate of 1.5 mm min^−1^. The compressive modulus (*E*), compressive strength (*σ*), and toughness were extracted from the resulting stress–strain curves. *E* was determined from the initial linear region (≤10% strain), while *σ* was defined as the stress at 85% strain. Toughness was calculated based on the integration of the stress–strain curve up to 85% strain. All mechanical properties were calculated using a custom MATLAB script. Compression testing was also performed on select mineralized scaffolds (*n* = 3) to evaluate the effects of 1 day and 2 weeks of exposure to 1X SBF.

### 2.12 | Statistical Analysis

All quantitative data were obtained from at least triplicate measurements (*n* ≥ 3) and are presented as mean ± standard deviation. Statistical analyses were performed using GraphPad Prism 10 (GraphPad Software, Inc., San Diego, CA, USA). One-way analysis of variance (ANOVA) followed by Tukey’s honestly significant difference (HSD) *post hoc* test was used for multiple comparisons. Statistical significance was defined as *p* < 0.05.

## 3 | Results and Discussion

### 3.1 | Scaffold Fabrication and Morphology

Hybrid composite scaffolds (i.e., with BG and CS) as well as BG-only, CS-only, and PCL-only scaffolds were evaluated to investigate how these components, collectively versus individually, influences properties (Figure 1; Table S1). Hybrid composite scaffolds were prepared by combining PCL-DA and CS-*graft*-PCL (1:24 molar ratio) at 100:0, 90:10, and 75:25 wt% (coinciding to 0, 1, and 2.5 wt% CS, respectively), and each with 0, 5, and 10 wt% BG. The combination of cross-linkable PCL-DA and thermoplastic CS-*graft*-PCL yielded semi-IPNs. The hybrid composite scaffolds (90:10%–5%, 90:10%–10%, 75:25%–5%, and 75:25%–10%) and BG-only composite scaffolds (PCL-5% and PCL-10%) were fabricated using a modified SCPL method with a fused salt/BG template that concentrates BG (5 or 10 wt%) on the pore walls [15]. To serve as non-composite controls, analogous CS-only scaffolds (90:10%–0% and 75:25%–0%) [19] and the PCL-only control (PCL-0%) scaffold were likewise prepared but with fused salt-only templates (i.e., no BG).

**FIGURE 2 |:**
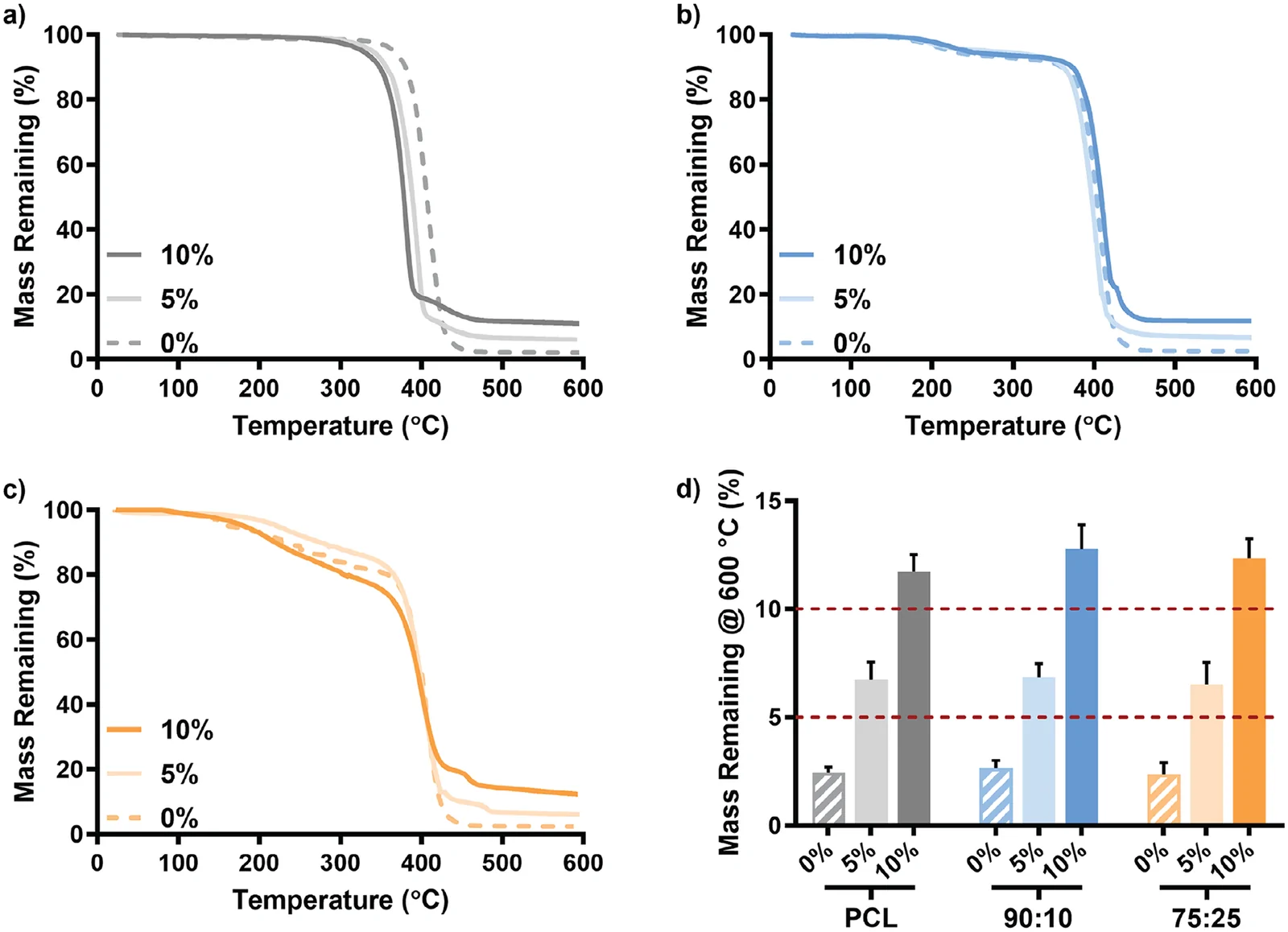
Representative TGA thermograms of (a) PCL, (b) 90:10, and (c) 75:25 scaffolds, (d) TGA plateau values at 600°C. Percentages refer to wt% of BG incorporated into each scaffold. (Note: PCL data previously reported [15]; 90:10%–0% and 75:25%–0% data previously reported [19]).

Adequate cross-linking of UV-curable PCL-DA during scaffold fabrication was confirmed by sol content measurements (Figure S1, Table S2). PCL-only scaffolds exhibited sol content values below ~5%, indicating effective cross-linking (> ~95%) of the PCL-DA, independent of BG inclusion. For PCL/CS semi-IPN scaffolds, sol content values aligned with the amount of non-cross-linkable CS-*graft*-PCL. In this way, for 90:10 compositions, sol contents did not exceed ~14%, whereas 75:25 compositions displayed expectedly higher sol contents (~23%–26%). Collectively, these results indicate that neither CS nor BG significantly interfered with PCL network cross-linking during scaffold fabrication. TGA further confirmed that target wt% ratios of PCL to CS-*graft*-PCL (100:0, 90:10, or 75:25) were achieved (Figure 2a–c). The onset of thermal degradation of CS-*graft*-PCL copolymers (~250–300°C) occurred earlier than that of cross-linked PCL networks (>400°C). Correspondingly, mass loss below 400°C was ~10% for the 90:10 compositions and ~25% for the 75:25 compositions, consistent with the intended CS-*graft*-PCL content in the PCL/CS semi-IPN scaffolds. The incorporation of BG wt% at near-targeted levels was subsequently verified via TGA plateau values (Figure 2d; Table S3).

**FIGURE 3 |:**
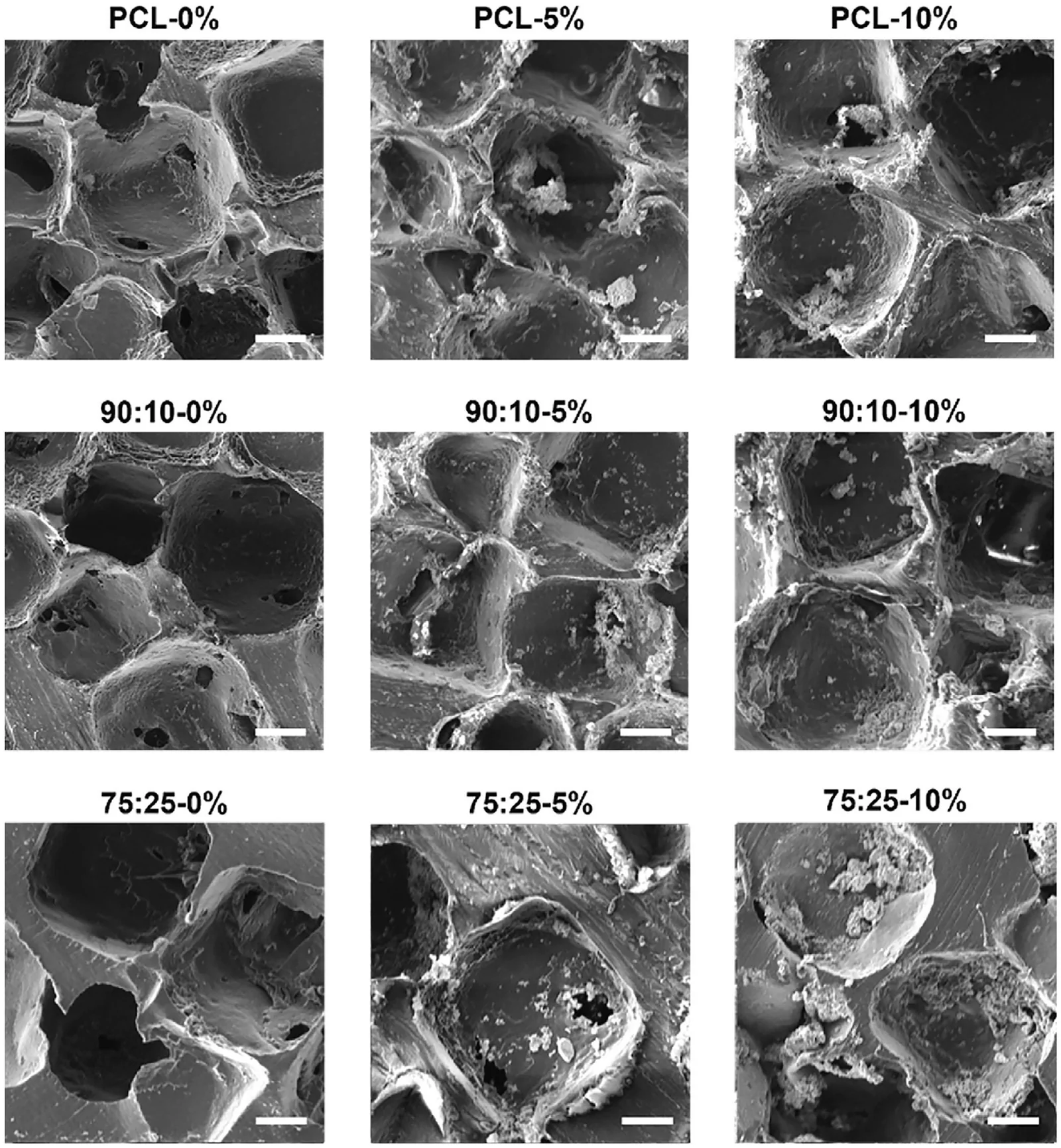
SEM images of scaffold cross sections. For composite scaffolds, prepared with 5 and 10 wt% BG, the BG was observed to be concentrated on the pore wall surfaces rather than within the pore struts. Scale bars = 100 μm.

To generate interconnected macropores critical to bone regeneration [26], scaffolds were readily fabricated via SCPL using fused salt/BG templates (for composites) and fused salt-only templates (for non-composites). SEM images of scaffold cross sections were used to assess pore size and morphology (Figure S2), as well as the presence and distribution of BG (Figure 3). Analysis confirmed successful NaCl leaching from composite and non-composite controls. Scaffold pore size remained largely unchanged (~240–255 μm) regardless of CS or BG incorporation (Figure S3a, Table S4), falling within the optimal range (200–300 μm) for osteogenesis and neotissue infiltration [26, 27]. All scaffolds exhibited high porosity (~60%–65%) (Figure S3b, Table S4) and good pore interconnectivity (~45%–50%) (Figure S3c, Table S4), both of which are expected to support osteogenesis [28, 29]. Furthermore, BG was observed to be concentrated on the pore walls, consistent with our prior report [15].

### 3.2 | Thermal Properties

**FIGURE 4 |:**
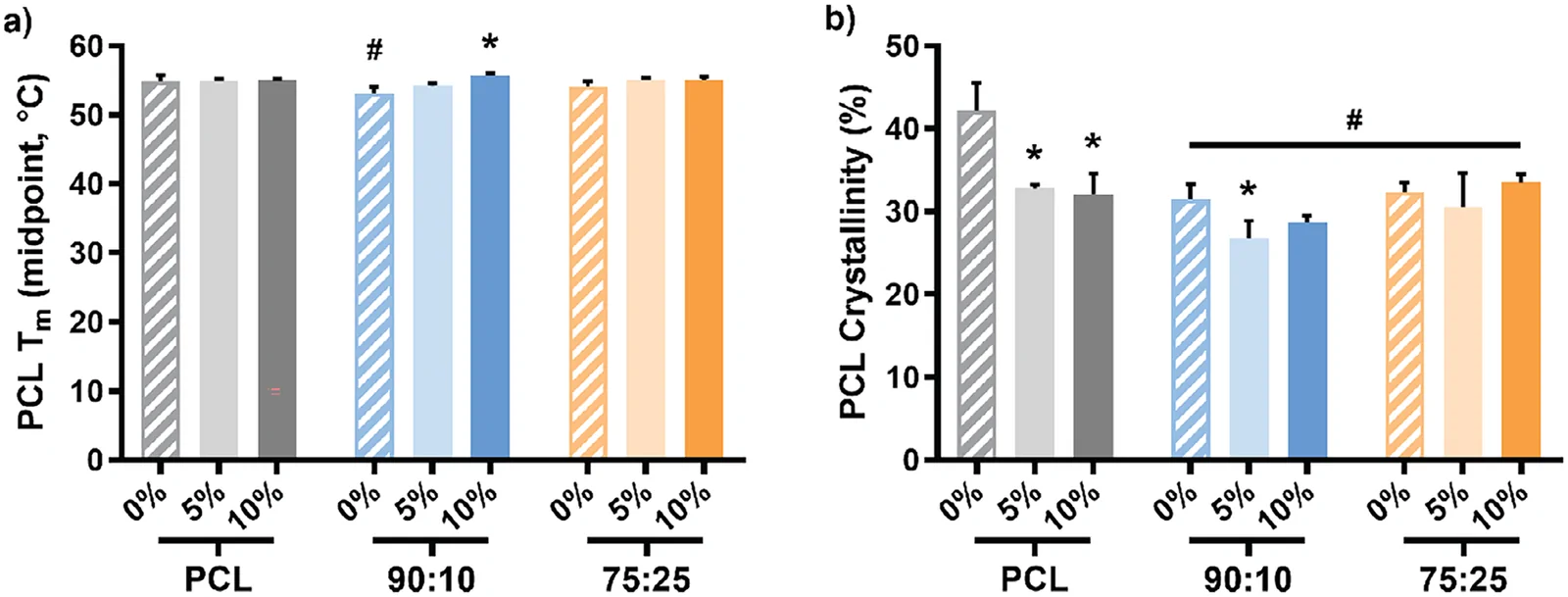
(a) PCL *T*_m_ and (b) PCL % crystallinity within scaffolds. Percentages refer to wt% of BG incorporated into each scaffold. * *p* < 0.05 vs. analogous scaffold with 0% BG; # *p* < 0.05 vs. PCL-0% (control). (Note: PCL data previously reported [15]; 90:10%–0% and 75:25%–0% data previously reported [19]).

The PCL *T*_m_ (i.e., *T*_trans_) represents the “self-fitting” temperature of the SMP scaffold within a defect, such that heating (*T* > *T*_trans_) affords shape recovery for conformal expansion and subsequent cooling (*T* < *T*_trans_) induces shape fixation. Beyond PCL crystalline lamellae serving as switching segments, PCL % crystallinity also influences both degradation and mechanical properties [30]. Hybrid composite scaffolds that include both CS (1 or 2.5 wt%) and BG (5 or 10 wt%) may uniquely impact PCL crystallinity. Thus, DSC was used to determine the *T*_m_ and % crystallinity of PCL within the scaffolds (Figure 4; Figure S4, Table S5). As observed previously, the individual inclusion of BG or CS led to retention of *T*_*m*_ values but reduction in PCL % crystallinity [15, 19]. When BG was introduced, PCL-5% and PCL-10% exhibited similar *T*_m_ values as PCL-0% (~55°C), but % crystallinity decreased from ~42% to ~32%–33%. This was consistent with prior reports in which various bioceramics reduced PCL crystallinity while *T*_m_ was maintained [31, 32]. In the absence of BG, PCL/CS scaffolds (90:10%–0% and 75:25%–0%) likewise maintained similar *T*_m_ values (~55°C) and decreased % crystallinity (~32%). This behavior has previously been reported for PCL/CS blends (CS < ~20 wt%) [33], where reduced crystallinity may be attributed to the formation of a miscible phase between PCL and CS, driven by intermolecular hydrogen bonding between PCL carbonyl groups and CS hydroxyl and amine groups [19], as well as differences in molecular mobility between the two polymers [33, 34]. In the case of hybrid composite scaffolds (90:10%–5%, 90:10%–10%, 75:25%–5%, and 75:25%–10%), the combination of BG and CS could potentially impact PCL crystallinity differently. However, the values of *T*_m_ (~54–56°C) and % crystallinity (~27%–33%) of these hybrid composite scaffolds were largely similar to those of “BG-only” or “CS-only” scaffolds. This may be attributed to the low CS content (1 and 2.5 wt%) that does not appreciably alter interaction of BG with PCL chains.

### 3.3 | Shape Memory Behavior

Hybrid composite scaffolds, with a combination of CS and BG, must retain excellent shape memory behavior to serve as “self-fitting” scaffolds. Thus, shape memory behavior of all scaffolds was quantitatively evaluated in terms of shape recovery (*R*_*r*_) and shape fixity (*R*_*f*_) using a previously developed method [22]. This utilized a circular model defect (*d* ~ 5 mm), representative of the bilateral rat calvarial defect commonly used for preliminary bone regeneration studies [35, 36]. The following process was completed for two cycles to remove scaffold thermal history. Scaffold specimens (*d* ~ 6 mm) were sequentially heated in ~60°C saline (*T* > *T*_trans_), press-fit into the defect, cooled to RT (*T* < *T*_trans_), and removed to determine *R*_*f*_. Next, *R*_*r*_ was determined by removing from the detect and returning to the warm saline for ~1 min. As previously described, covalent cross-links of the PCL network act as netpoints that define the permanent scaffold shape, while PCL crystalline lamellae function as switching segments that enable shape actuation above and below *T*_m_. Thus, sufficient PCL network cross-linking and crystallinity are expected to maintain shape memory behavior. Versus PCL-0%, all scaffolds exhibited sufficiently cross-linked PCL networks, as indicated by low sol content values (Figure S1, Table S2). Values of PCL *T*_m_ remained generally similar (~53–56 °C), whereas crystallinity was reduced to ~27%–33% versus PCL-0% (~42%) (Figure 4b; Table S5). Nevertheless, hybrid composite scaffolds demonstrated excellent shape fixity (*R*_*f*_) and shape recovery (*R*_*r*_) values (≥ ~98%) (Table S6), comparable to the PCL-0% as well as the BG-only and CS-only scaffolds reported previously [15, 19]. Thus, the % crystallinity is sufficient to serve as effective switching segments, confirming that the shape memory behavior remains unaffected by the incorporation of both CS and BG, and enabling reliable “self-fitting” behavior during implantation.

### 3.4 | Degradation

**FIGURE 5 |:**
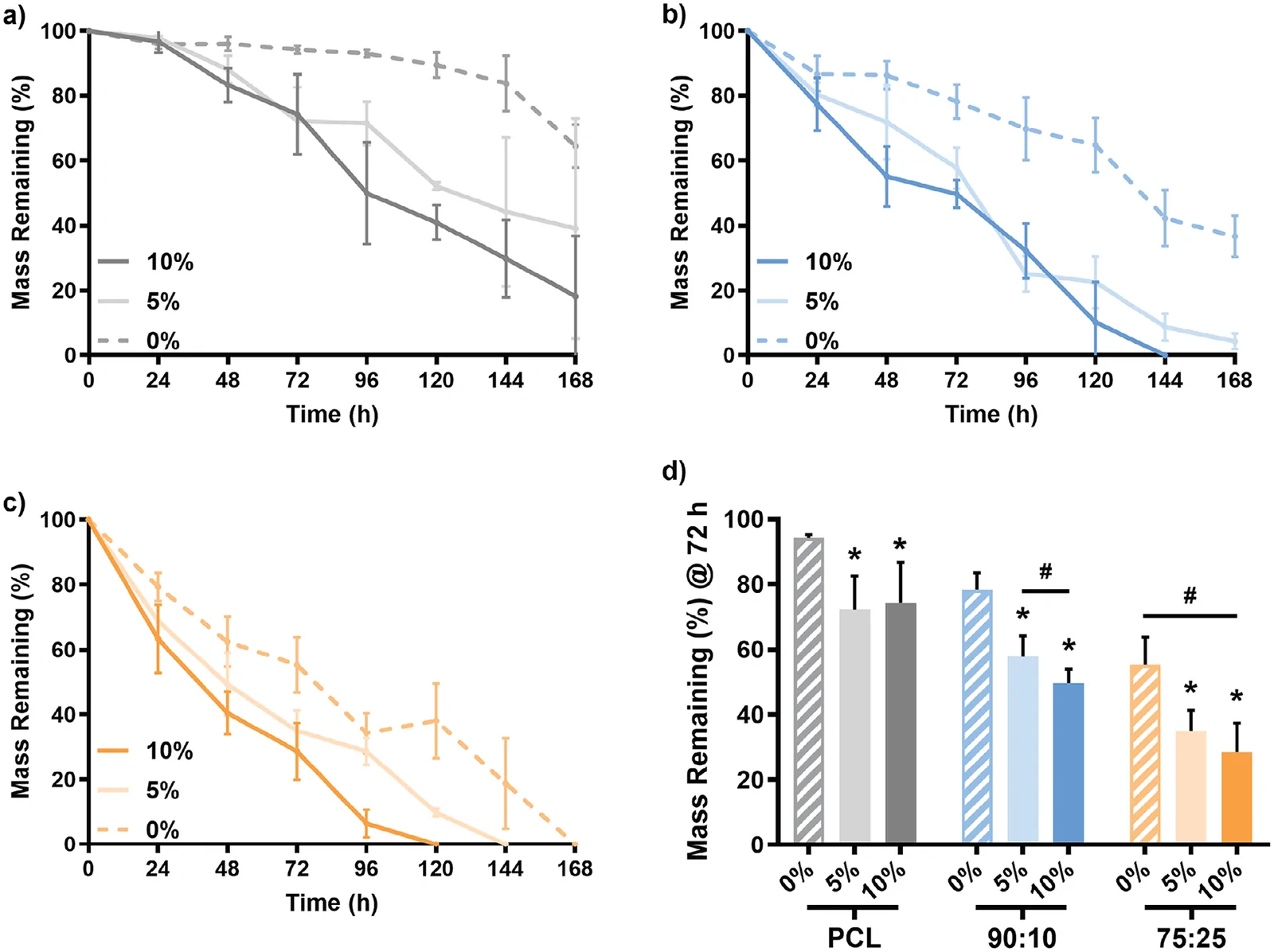
Gravimetric mass loss over 7 days (0.2 m NaOH, 37°C, 60 rpm) for (a) PCL, (b) 90:10, and (c) 75:25 scaffolds, (d) Scaffold mass remaining at 72 h. Percentages refer to wt% of BG incorporated into each scaffold. * *p* < 0.05 vs. analogous scaffold with 0% BG; $ *p* < 0.05 vs. analogous hybrid composite scaffolds (i.e., 5 vs. 10 wt% BG); # *p* < 0.05 vs. PCL-0% (control). (Note: PCL data previously reported [15]).

Scaffolds with degradation rates that align with neotissue formation are expected to enhance defect healing [37]. Thus, given the slow degradation of PCL (~2 years) relative to neotissue formation (~2–3 months), PCL-0% scaffolds may limit bone regeneration [38, 39], Increasing the degradation rate of PCL-based scaffolds can be achieved through various strategies that enhance water uptake and, subsequently, hydrolysis [9]. We have previously demonstrated that reducing PCL crystallinity accelerates degradation in PCL SMP scaffolds [30]. Similarly, semi-IPNs composed of PCL and poly(l-lactic acid) (PLLA) degrade significantly faster than the corresponding PCL network scaffolds, due to polymer phase separation and enhanced water permeability [21, 22]. The incorporation of bioceramics also enhances degradation in composite scaffolds through increased hydrophilicity [13]. In line with these findings, hybrid CS/PCL scaffolds [19] and BG-based composite scaffolds [15] demonstrated accelerated degradation versus PCL-only scaffolds owing to increased hydrophilicity as well as phase separation for CS/PCL scaffolds. Reduced PCL crystallinity (~27%–33%), combining enhanced hydrophilicity and phase separation, therefore suggests that PCL/CS/BG hybrid composite scaffolds would degrade significantly faster than corresponding BG-only or CS-only scaffolds. Thus, scaffold degradation behavior was assessed in vitro under base-catalyzed conditions (0.2 m NaOH, 37°C, 60 rpm) following an adapted ASTM F1635 protocol (Figure 5; Figure S5, Table S7). Indeed, hybrid composite scaffolds degraded faster than corresponding BG-only and CS-only scaffolds. Greater CS content led to faster degradation (Figure S5), while an increase in BG from 5% versus 10% BG did not lead to further enhancements (Figure 5a–c). Thus, degradation rates were the highest for 75:25%–5% and 75:25%–10% (Figure 5d). These results demonstrate the tunable degradation behavior of PCL/CS/BG hybrid composite scaffolds, driven by the synergistic effects of CS and BG.

### 3.5 | Bioactivity

**FIGURE 6 |:**
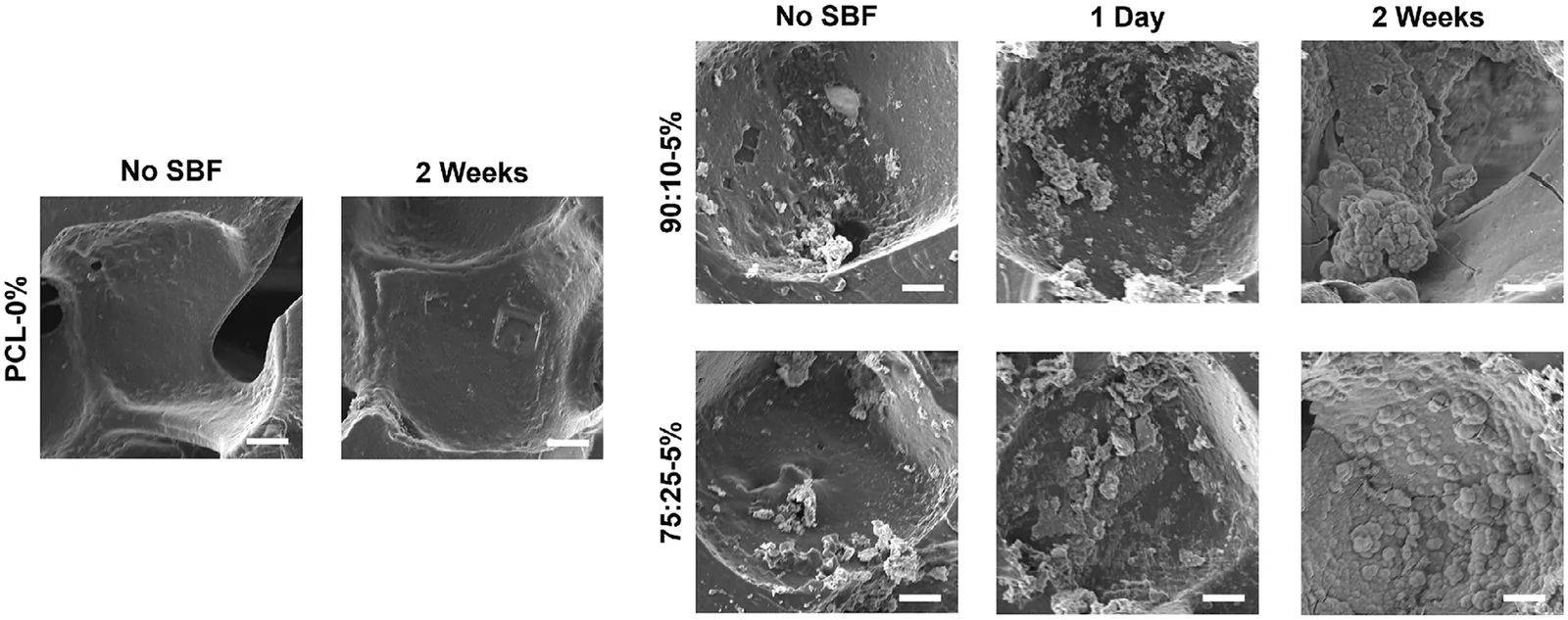
SEM images of PCL-only (PCL-0%) and hybrid composite (90:10%–5% and 75:25%–5%) scaffold cross sections prior to and after 1X SBF exposure. Scale bars = 50 μm.

The noted properties (e.g., porosity, shape memory behavior, and degradation) of the hybrid composite scaffolds are favorable for bone regeneration. However, bioactivity, specifically the ability to induce HAp mineralization, is critical for achieving effective osseointegration and promoting osteoinductivity [13, 40, 41]. Since BG-based composite scaffolds exhibited bioactivity while hybrid CS/PCL scaffolds did not, hybrid composite scaffolds that combined BG and CS were anticipated to be bioactive. While BG was included at just 5 and 10 wt%, localization on scaffold pore walls (Figure 3) maximized its contact with the surrounding environment. Scaffold bioactivity was assessed by exposure to 1X SBF [25]. After just 1 day of SBF exposure, all BG-containing composite scaffolds, regardless of CS content, showed mineralization on scaffold pores (Figure 6; Figure S6). After 2 weeks, a greater degree of mineralization was observed (Figure 6; Figure S7). Again, this points to the potency of BG localized on pore wall surfaces, despite low concentrations. As expected, BG-free PCL (PCL-0%) and PCL/CS (90:10%–0% and 75:25%–0%) scaffolds showed no signs of mineralization. To confirm the observed mineralization as HAp, EDS was used to determine Ca/P molar ratios characteristic of BG (Ca/p = 5) [42] and HAp (Ca/p = 1.67) [43] on composite scaffolds containing CS (75:25%–5%) (Figure S8). For these composites, the Ca/P molar ratio increased from ~1.49 after 1 day to ~1.67 after 2 weeks of SBF exposure (Figure S8c,d), consistent with HAp formation. Collectively, these results demonstrate the successful formation of bioactive PCL/CS/BG hybrid composite scaffolds capable of inducing HAp mineralization.

### 3.6 | Compressive Mechanical Properties

The utility of PCL/CS/BG SMP scaffolds (90:10%–5%, 90:10%–10%, 75:25%–5%, and 75:25%–10%) for healing CMF bone defects is dependent on mechanical robustness [44, 45]. Paramount is the retention of favorable properties of the PCL-only (PCL-0%) scaffold, particularly rigidity in the range of trabecular bone and lack of brittleness [46]. The independent incorporation of BG [15] and CS [19] was previously shown to afford mechanically robust scaffolds. For BG-based composite scaffolds, this was attributed to low levels of BG (5 and 10 wt%) concentrated on pore walls, as opposed to higher concentrations and presence within pore walls [15]. While pristine CS scaffolds exhibit poor mechanical properties (e.g., low strength) [47], low levels of CS (<2.5 wt%) afforded CS/PCL scaffolds with mechanical properties similar to the PCL-only scaffold [19]. Thus, PCL/CS/BG hybrid composite scaffolds were likewise expected to retain mechanical robustness.

**FIGURE 7 |:**
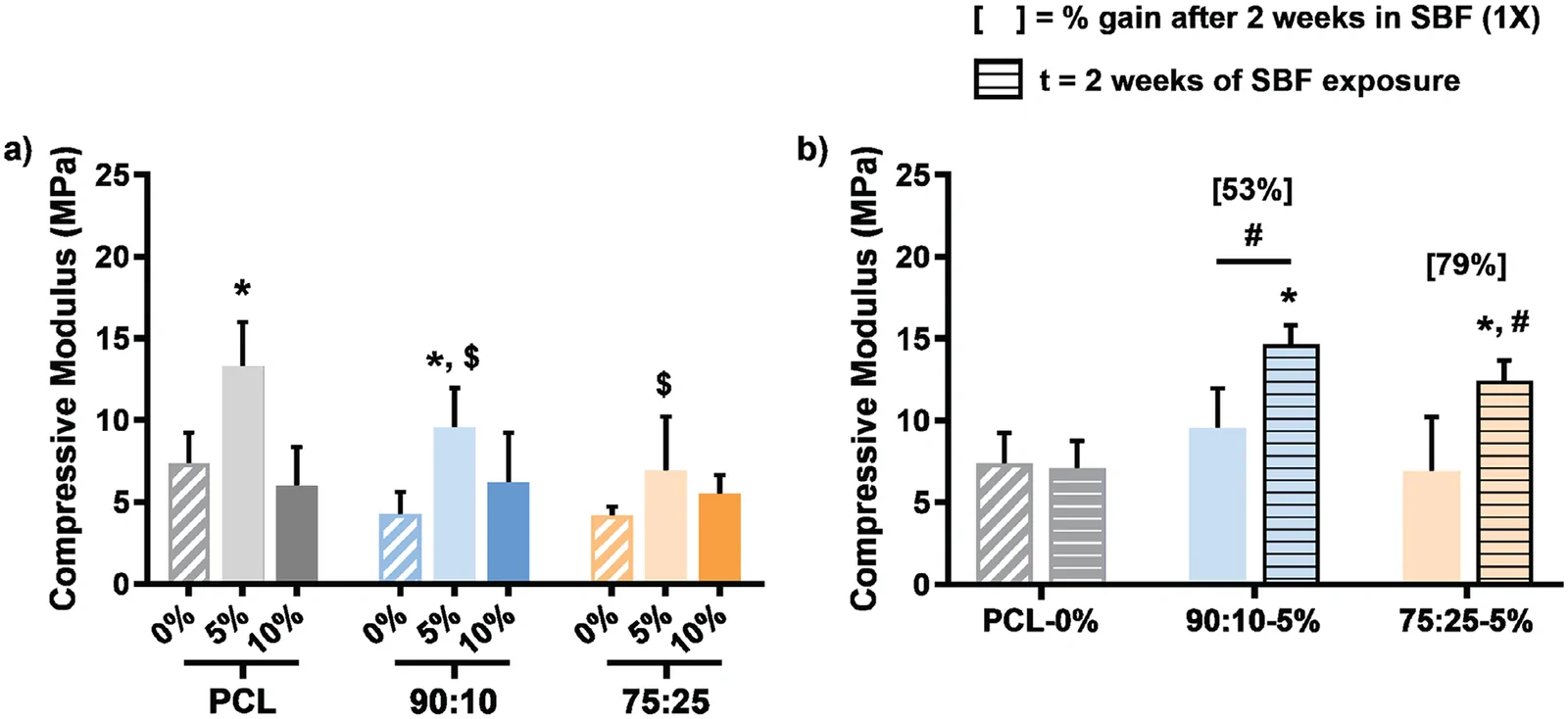
(a) Compressive modulus (*E*) values of PCL-only (PCL-0%), BG-only (PCL-5% and PCL-10%), CS-only (90:10%–0% and 75:25%–0%), and hybrid composite (90:10%–5%, 90:10%–10%, 75:25%–5%, and 75:25%–10%) scaffolds. * p < 0.05 vs. PCL-0% (control); $ *p* < 0.05 vs. PCL scaffolds with same wt% BG; (b) *E* values of PCL-0% and hybrid composite scaffolds with 5 wt% BG (90:10%–5% and 75:25%–5%) before and after (t = 2 weeks) 1X SBF exposure. * *p* < 0.05 vs. analogous pre-mineralized scaffold; # *p* < 0.05 vs. pre-mineralized PCL-0% (control). Percentages refer to wt% of BG incorporated into each scaffold. (*Note:* pre-mineralized PCL-0% data previously reported [15]).

The extent of PCL network formation will impact scaffold mechanical properties. As noted, hybrid composite scaffolds exhibited highly cross-linked PCL networks as indicated by low sol content values, similar to that of the PCL-only control (PCL-0%), BG-only (PCL-5%, PCL-10%), and CS-only (90:10%–0%, 75:25%–0%) scaffolds (Figure S1, Table S2). However, relative to the PCL-only control (~42%), the % crystallinity was reduced for the hybrid composite scaffolds (~27%–33%) as well as BG-only (~33%) and CS-only (~32%) scaffolds (Figure 4; Table S5). While shape memory properties were retained (Table S6), a reduction in PCL % crystallinity may diminish scaffold rigidity (i.e., modulus, *E*). Thus, compression testing was conducted to determine *E* values of scaffolds (Figure 7a; Table S8). Versus PCL-0% (*E* ~ 7.4 MPa), the inclusion of 5 wt% BG led to an increase in *E* (PCL-5%, *E* ~ 13 MPa) while scaffolds with 10 wt% BG produced similar *E* values (PCL-10%, *E* ~ 6 MPa). Given that analogous PCL scaffolds prepared with >10 wt% BG exhibited relatively lower *E* values stemming from loss of pore wall integrity [15], the superior performance with 5 wt% BG may be indicative of optimal reinforcement. For scaffolds with 1 wt% CS (90:10%–0%) and 2.5 wt% CS (75:25%–0%), *E* values were similar to PCL-0% [19]. In the case of hybrid composite scaffolds, only 90:10%–5% (*E* ~ 9.6 MPa) achieved a higher *E* versus PCL-0%, although it was lower than that of PCL-5%. All other hybrid composites had *E* values similar to PCL-0%, including 75:25%–5% (*E* ~ 7 MPa). The greater *E* of 90:10%–5% versus 75:25%–5%, despite having similar % crystallinity, may indicate that a PCL-based matrix with a slightly lower CS affords superior interfacial bond strength with the BG. Similar trends were observed with compressive strength (*σ*, measured at 85% strain), with the hybrid composite scaffold 90:10%–5% (*σ* ~ 43 MPa) displaying higher values versus PCL-0% (*σ* ~ 32 MPa) but similar values to PCL-5% (*σ* ~ 50 MPa) (Table S8, Figure S9a). All other compositions, including the other hybrid composite scaffolds, exhibited *σ* values similar to PCL-0%. All hybrid composite scaffolds likewise withstood fracture at ≤85% strain, and exhibited similar toughness to PCL-0% (Table S8, Figure S9b).

HAp mineralization of a bioactive scaffold may lead to an increase in *E* [48–51], which is expected to afford additional mechanical support during bone tissue healing. As noted above, all hybrid composite scaffolds exhibited HAp mineralization after just 1 day in 1X SBF (Figure 6; Figure S6), and levels notably increased after 2 weeks (Figure 6; Figure S7). This behavior was likewise observed in the BG-only scaffolds (PCL-5% and PCL-10%), but absent in PCL-0% and the CS-only scaffolds (90:10%–0% and 75:25%–0%). Based on the overall superior *E* values of scaffolds with 5% BG, the corresponding post-mineralization *E* values were assessed for 90:10%–5% and 75:25%–5%, and compared with the ‘non-bioactive’ PCL-0% (Figure 7b; Table S9). Despite HAp mineralization observed at 1 day, there was no corresponding significant increase in hybrid composite scaffold *E*. However, after 2 weeks, post-mineralized *E* values increased for both 90:10%–5% (~14.7 MPa; ~53% increase) and 75:25%–5% (~12.4 MPa; ~79% increase). These values notably surpass that of PCL-0% (~7.1 MPa). HAp mineralization resulted in the retention of strength and toughness of these hybrid composite scaffolds, with a slight increase in *σ* (~12%) observed for 90:10%–5%.

## 4 | Conclusions

A conformally fitting, “off-the-shelf” scaffold capable of supporting regeneration through intrinsic, material-driven functionality remains a critical unmet need and represents a promising platform for CMF bone defect healing. We previously reported “self-fitting” SMP scaffolds, based on cross-linked PCL-DA networks, that enable conformal fitting of irregular defect geometries through shape recovery (i.e., thermally induced expansion). The lack of bioactivity and the slow rate of degradation were limitations of such PCL-only scaffolds. Subsequently, analogous composite scaffolds were formed by the incorporation of BG, resulting in bioactivity and faster degradation. Hybrid scaffolds (i.e., formed from a synthetic and naturally derived polymer) were also prepared that included CS, resulting in antimicrobial activity as well as enhanced degradation rates. Herein, toward developing “self-fitting” scaffolds with robust bioactivity and further enhanced degradation rates, we developed hybrid composites scaffolds based on the inclusion of both BG and CS. Four PCL/CS/BG scaffolds were formed as semi-IPNs by combining cross-linked PCL-DA with non-cross-linked CS-*graft*-PCL (1:24 molar ratio) at 90:10 and 75:25 wt%, resulting in 1 and 2.25 wt% CS, respectively, and each with BG loadings of 5 or 10 wt%. PCL-only, BG-only, and CS-only scaffolds served as controls to elucidate the combined effects of CS and BG on hybrid composite scaffold properties. Successful localization of BG on the composite scaffold pore walls was achieved using a modified SCPL protocol employing a fused salt/BG template, and TGA confirmed that BG loadings were near the targeted wt%. This approach also produced scaffolds with high porosity (~60%–65%) and interconnected macropores (~240–255 μm), both conducive to osteogenesis. While the values of PCL *T*_m_ remained generally similar (~53–56 °C), PCL % crystallinity of hybrid composite scaffolds was reduced to ~27%–33%, as for BG-only and CS-only scaffolds, versus the PCL-only scaffold (~42%). However, excellent shape memory behavior was retained, indicating sufficiency of this level of crystallinity (i.e., switching segments). Hybrid composite scaffolds also exhibited faster rates of degradation than corresponding BG-only and CS-only scaffolds. Thus, a synergistic contribution was afforded by the hydrophilicity imparted by CS and BG, as well as phase separation effects by CS. Notably, while degradation increased with increased CS content, an increase in BG from 5 to 10 wt% did not yield differences. Hybrid composite scaffolds, with 5 or 10 wt% BG and with both CS levels, exhibited HAp mineralization after just 1 day of exposure to 1X SBF (37 °C) and became more pronounced after 2 weeks. Hybrid composite scaffolds also exhibited robust mechanical properties, including the trabecular bone-like *E* and non-brittleness of the PCL-only scaffold. Most exhibited similar *E* and *σ* values to that of the PCL-only scaffold (*E* ~ 7.4 MPa; *σ* ~ 32 MPa), while one (90:10%–5%) achieved a higher value (*E* ~ 9.6 MPa; *σ* ~ 43 MPa). All hybrid composite scaffolds exhibited similar toughness of the control, and withstood fracture at ≤85% strain. Hybrid composite scaffolds with 5 wt% BG were also subjected to compressive testing following 2 weeks of HAp mineralization. The 90:10%–5% and 75:25%–5% scaffolds exhibited a ~53% and ~79% increase, respectively, to surpass that of the non-bioactive (i.e., no HAp mineralization) PCL-only scaffold. Overall, this work advances a materials-driven regenerative approach for CMF bone defect healing using PCL/CS/BG composite scaffolds with accelerated degradation and intrinsic bioactivity. Future studies will investigate the ability of these composites to support in vitro cellular mineralization, as well as their capacity to resist microbial colonization following BG incorporation.

## Supplementary Material

Supporting Info

Additional supporting information can be found online in the Supporting Information section.

**Supporting File:** mame70250-sup-0001-SuppMat.pdf.

## Data Availability

Raw data is available via the Texas Data Repository (TDR).
