# Supplementary material for: PCL/Chitosan/Bioglass Hybrid Composite Shape Memory Scaffolds With Accelerated Degradation and Enhanced Bioactivity
Source: Macromol Mater Eng. Author manuscript; Available in PMC 2026 Sep 10. (PMC13557040; doi:10.1002/mame.70250)
Supplement: Supplementary file 1 [file NIHMS2207304-supplement-Supporting_Info.pdf]

Supporting Information

PCL/Chitosan/Bioglass Hybrid Composite Shape Memory Scaffolds with Accelerated Degradation and Enhanced Bioactivity

Damion T. Dixon[1,], Darian K. Kanu[2], and Melissa A. Grunlan[1,2,3,*]*

[1]Department of Biomedical Engineering, Texas A&M University, College Station, Texas 77843, United States

[2]Department of Materials Science and Engineering, Texas A&M University, College Station, Texas 77843, United States

[3]Department of Chemistry, Texas A&M University, College Station, Texas 77843, United States

Corresponding Authors:

Damion T. Dixon
Email: dtd@tamu.edu

Melissa A. Grunlan
Email: mgrunlan@tamu.edu

19 Pages

10 Figures

9 Tables

Table S1. Scaffold compositions and notations.

Scaffold Notation	Bioglass (wt%) (w.r.t. total macromer)	CS-*graft*-PCL Incorporation (wt%)	Chitosan Content[a] (wt%)
PCL-0%	0	0	0
PCL-5%	5	0	0
PCL-10%	10	0	0
90:10-0%	0	10	0.99 ± 0.04
90:10-5%	5	10	0.99 ± 0.04
90:10-10%	10	10	0.99 ± 0.04
75:25-0%	0	25	2.50 ± 0.10
75:25-5%	5	25	2.50 ± 0.10
75:25-10%	10	25	2.50 ± 0.10

[a]Determined via TGA of scaffolds. (Note: Chitosan content data for 90:10 and 75:25 scaffolds previously reported [1]).

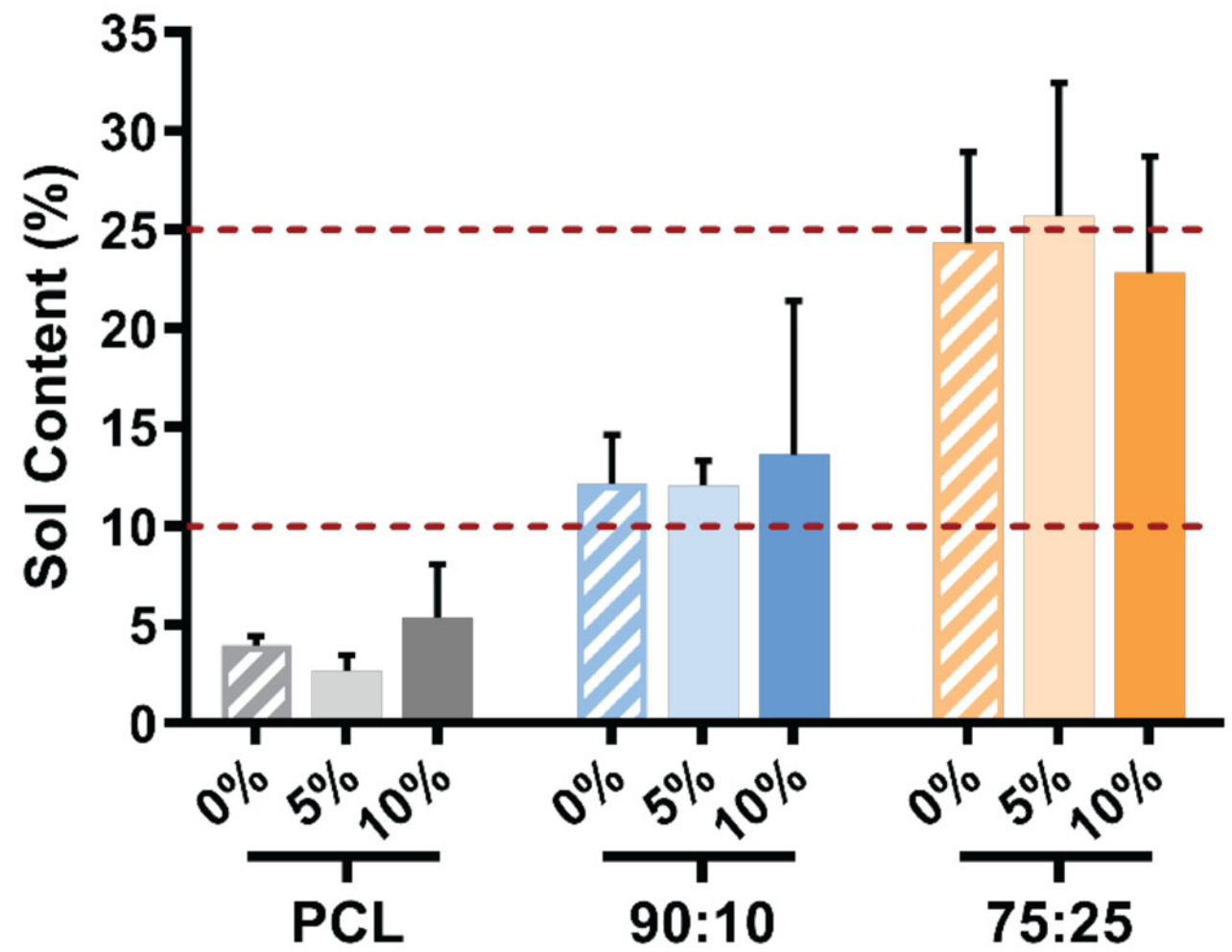

Figure S1. Sol content (% mass loss) of scaffolds. "Dashed" lines (10 or 25%) correspond to wt% non-cross-linkable CS-*graft*-PCL formulated into scaffolds. Percentages refer to wt% of BG incorporated into each scaffold.

Table S2. Scaffold sol content (% mass loss) values. Percentages refer to wt% of BG incorporated into each scaffold.

Scaffold	Sol Content (%)
PCL-0%	3.96 ± 0.45
PCL-5%	2.64 ± 0.83
PCL-10%	5.36 ± 2.71
90:10-0%	12.11 ± 2.71
90:10-5%	12.03 ± 1.26
90:10-10%	13.57 ± 7.83
75:25-0%	24.30 ± 4.66
75:25-5%	25.67 ± 6.77
75:25-10%	22.80 ± 5.91

Table S3. TGA wt% plateau values at 600 °C indicating BG incorporation. Percentages refer to wt% of BG incorporated into each scaffold. (Note: PCL data previously reported [2]).

Scaffold	Mass Remaining (%) at 600 °C
PCL-0%	2.44 ± 0.25
PCL-5%	6.74 ± 0.80
PCL-10%	11.73 ± 0.78
90:10-0%	2.65 ± 0.36
90:10-5%	6.83 ± 0.65
90:10-10%	12.76 ± 1.12
75:25-0%	2.36 ± 0.53
75:25-5%	6.51 ± 1.02
75:25-10%	12.35 ± 0.91

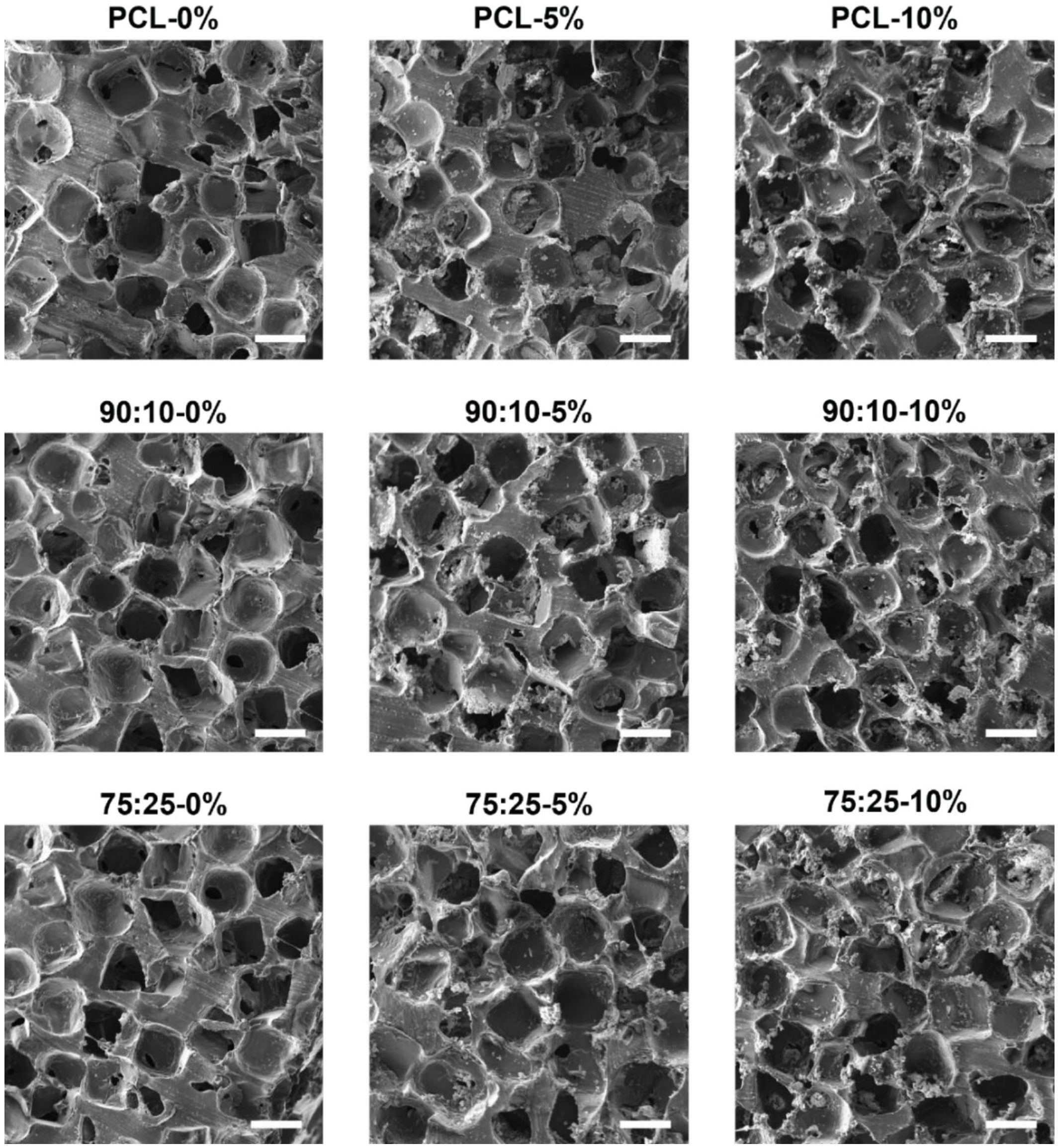

Figure S2. SEM images of scaffold cross sections. Percentages refer to wt% of BG incorporated into each scaffold. Scale bars = 200 μm.

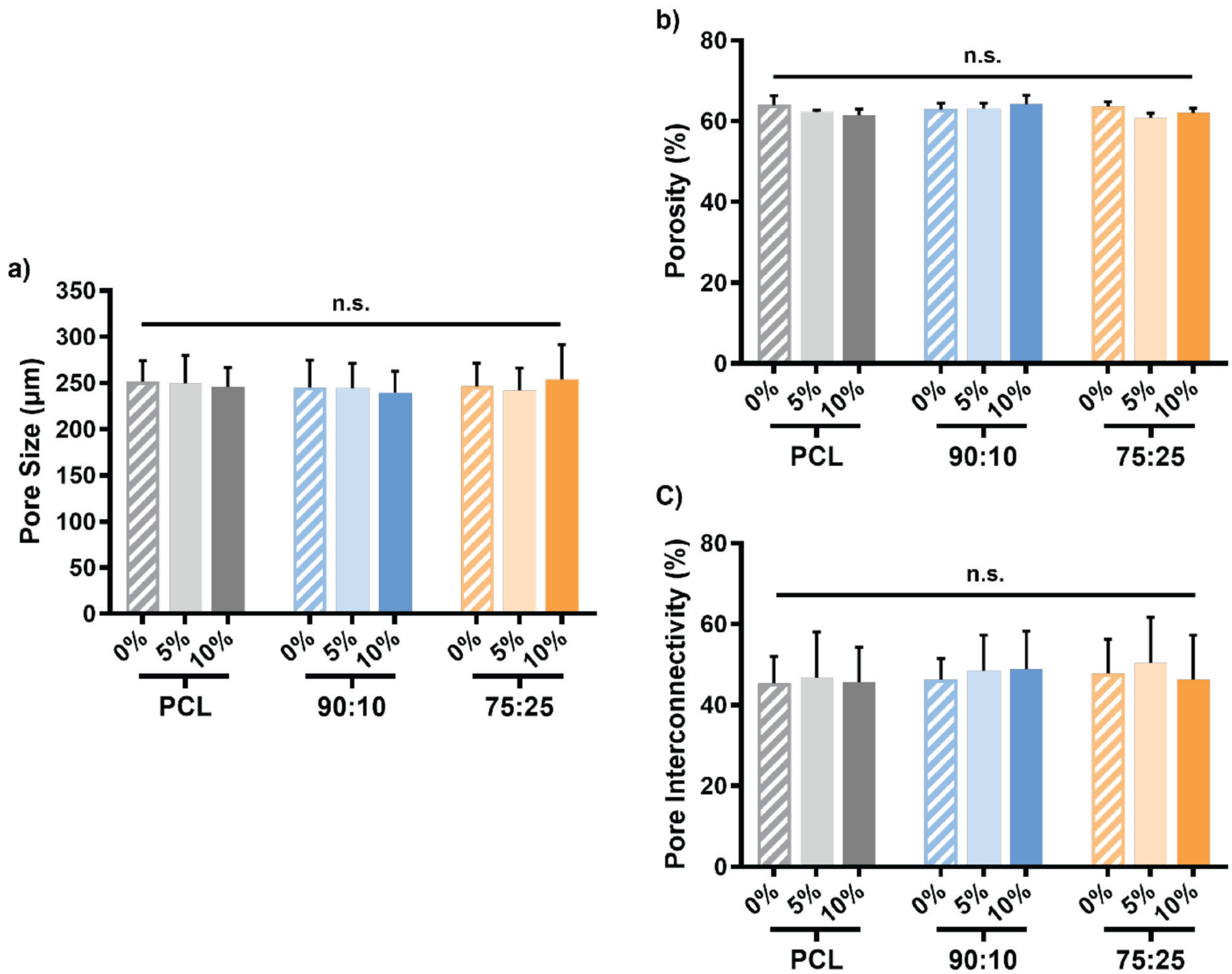

Figure S3. (a) Pore size, (b) % porosity, and (c) % pore interconnectivity of scaffolds. Percentages refer to wt% of BG incorporated into each scaffold. *n.s.* = no statistical significance.

Table S4. Scaffold pore size, % porosity, and % pore interconnectivity. Percentages refer to wt% of BG incorporated into each scaffold.

Scaffold	Pore Size (µm)	Porosity (%)	Pore Interconnectivity (%)
PCL-0%	251.64 ± 22.36	63.95 ± 2.32	45.32 ± 6.65
PCL-5%	249.36 ± 30.52	62.25 ± 0.45	46.78 ± 11.25
PCL-10%	245.63 ± 21.01	61.38 ± 1.56	45.70 ± 8.25
90:10-0%	244.98 ± 29.25	62.89 ± 1.52	46.23 ± 5.28
90:10-5%	243.98 ± 26.89	63.07 ± 1.36	48.35 ± 8.88
90:10-10%	239.67 ± 22.54	64.12 ± 2.25	48.82 ± 9.44
75:25-0%	245.95 ± 25.25	63.58 ± 1.20	47.75 ± 8.50
75:25-5%	241.56 ± 24.28	60.81 ± 1.14	50.32 ± 11.41
75:25-10%	253.92 ± 37.39	62.02 ± 1.13	46.20 ± 11.05

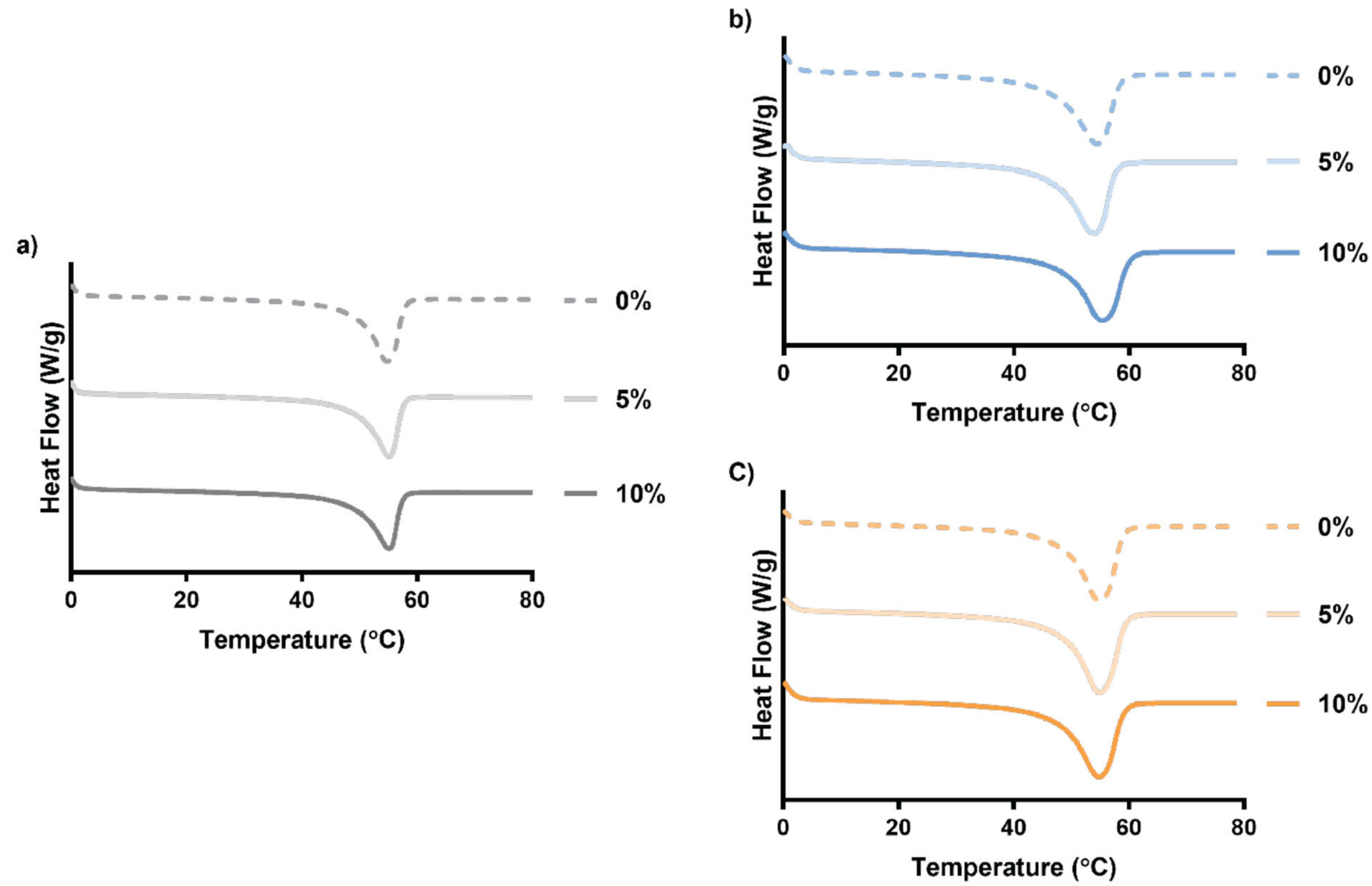

Figure S4. Representative DSC thermograms of (a) PCL, (b) 90:10, and (c) 75:25 scaffolds. Percentages refer to wt% of BG incorporated into each scaffold. (Note: PCL data previously reported [2]; 90:10-0% and 75:25-0% data previously reported [1]).

Table S5. Scaffold PCL T_m and PCL % crystallinity. Percentages refer to wt% of BG incorporated into each scaffold. (Note: PCL data previously reported [2]; 90:10-0% and 75:25-0% data previously reported [1]).

Scaffold	PCL T_m (midpoint, °C)	PCL Crystallinity (%)
PCL-0%	54.82 ± 0.91	42.20 ± 3.32
PCL-5%	54.88 ± 0.27	32.80 ± 0.41
PCL-10%	54.96 ± 0.21	32.09 ± 2.53
90:10-0%	53.11 ± 1.02	31.51 ± 1.78
90:10-5%	54.15 ± 0.36	26.79 ± 2.08
90:10-10%	55.69 ± 0.33	28.62 ± 0.85
75:25-0%	54.11 ± 0.75	32.40 ± 1.12
75:25-5%	55.12 ± 0.23	30.50 ± 4.16
75:25-10%	55.05 ± 0.49	33.48 ± 1.02

Table S6. Quantitative shape memory testing results. Percentages refer to wt% of BG incorporated into each scaffold.

Scaffold	Shape Fixity (R_f, %)	Shape Recovery (R_r, %)
PCL-0%	103.50 ± 2.37	100.25 ± 0.65
PCL-5%	99.62 ± 1.36	101.87 ± 0.30
PCL-10%	98.52 ± 2.44	101.88 ± 0.64
90:10-0%	98.21 ± 2.34	101.20 ± 0.75
90:10-5%	98.65 ± 0.63	99.05 ± 0.22
90:10-10%	102.75 ± 1.81	101.94 ± 1.66
75:25-0%	99.90 ± 1.55	97.52 ± 1.07
75:25-5%	99.65 ± 2.05	103.10 ± 1.16
75:25-10%	100.90 ± 0.35	102.25 ± 1.89

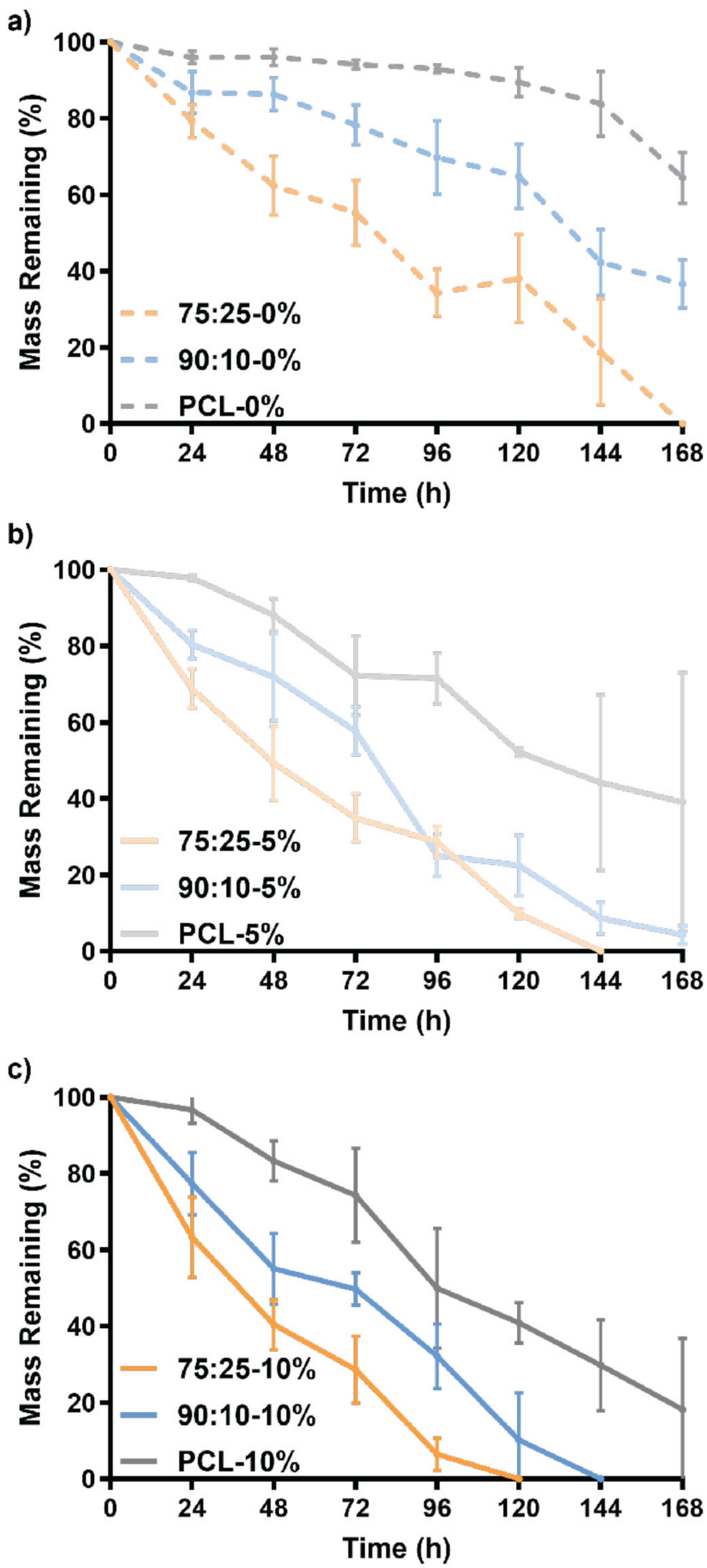

Figure S5. Gravimetric mass loss over 7 days (0.2 M NaOH, 37 °C, 60 rpm) for (a) 0% BG, (b) 5% BG, and (c) 10% BG scaffolds. (Note: PCL data previously reported [2]).

Table S7. Scaffold mass remaining (%) during accelerated (0.2 M NaOH, 37 °C, 60 rpm) degradation study. Percentages refer to wt% of BG incorporated into each scaffold. (Note: PCL data previously reported [2]).

Scaffold	24 hrs	48 hrs	72 hrs	96 hrs	120 hrs	144 hrs	168 hrs
PCL-0%	96.01 ± 1.69	96.08 ± 2.18	94.19 ± 1.20	93.02 ± 1.13	89.49 ± 3.79	83.80 ± 8.56	64.48 ± 6.64
PCL-5%	97.89 ± 0.68	88.05 ± 4.30	72.28 ± 10.32	71.46 ± 6.66	52.20 ± 1.14	44.23 ± 22.95	39.15 ± 33.92
PCL-10%	96.78 ± 2.52	83.30 ± 5.24	74.36 ± 12.37	49.98 ± 15.72	40.94 ± 5.35	29.76 ± 11.87	18.17 ± 18.61
90:10-0%	86.78 ± 5.52	86.34 ± 4.35	78.31 ± 5.22	69.76 ± 9.64	64.85 ± 8.44	42.25 ± 8.69	36.63 ± 6.37
90:10-5%	80.4 ± 3.67	71.88 ± 11.45	57.75 ± 6.36	25.14 ± 5.52	22.51 ± 7.94	8.69 ± 4.11	4.38 ± 2.38
90:10-10%	77.36 ± 8.24	55.10 ± 9.31	49.72 ± 4.25	32.15 ± 8.44	10.20 ± 12.34	0.00 ± 0.00	0.00 ± 0.00
75:25-0%	79.36 ± 4.34	62.46 ± 7.74	55.27 ± 8.54	34.25 ± 6.20	38.05 ± 11.51	18.82 ± 13.95	0.00 ± 0.00
75:25-5%	68.82 ± 5.12	49.26 ± 9.75	34.95 ± 6.32	28.65 ± 4.10	9.81 ± 1.25	0.00 ± 0.00	0.00 ± 0.00
75:25-10%	63.36 ± 10.54	40.44 ± 6.64	28.65 ± 8.78	6.50 ± 4.30	0.00 ± 0.00	0.00 ± 0.00	0.00 ± 0.00

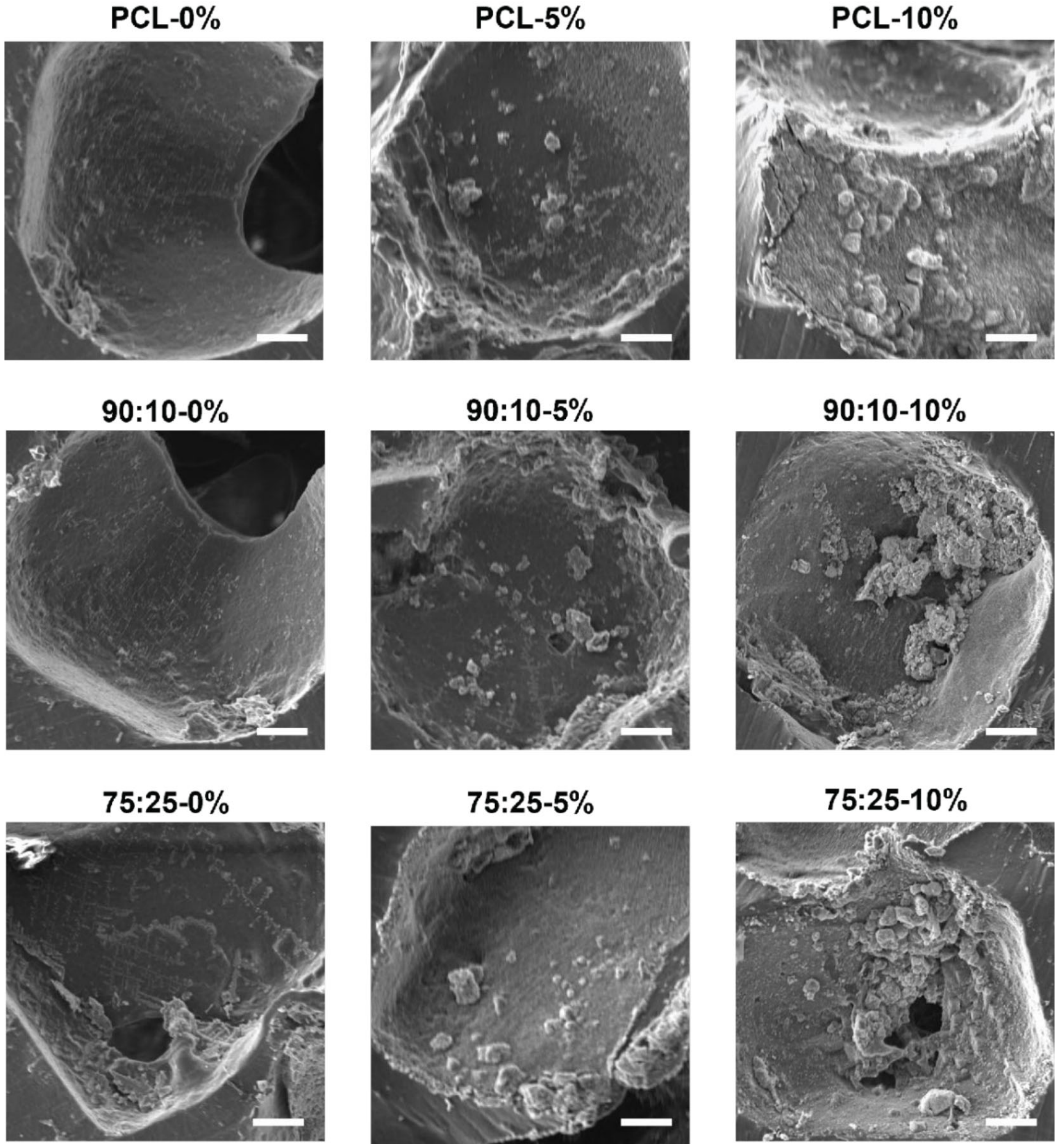

Figure S6. SEM images of scaffold cross sections after 1 day of 1X SBF exposure. Percentages refer to wt% of BG incorporated into each scaffold. Scale bars = 50 µm.

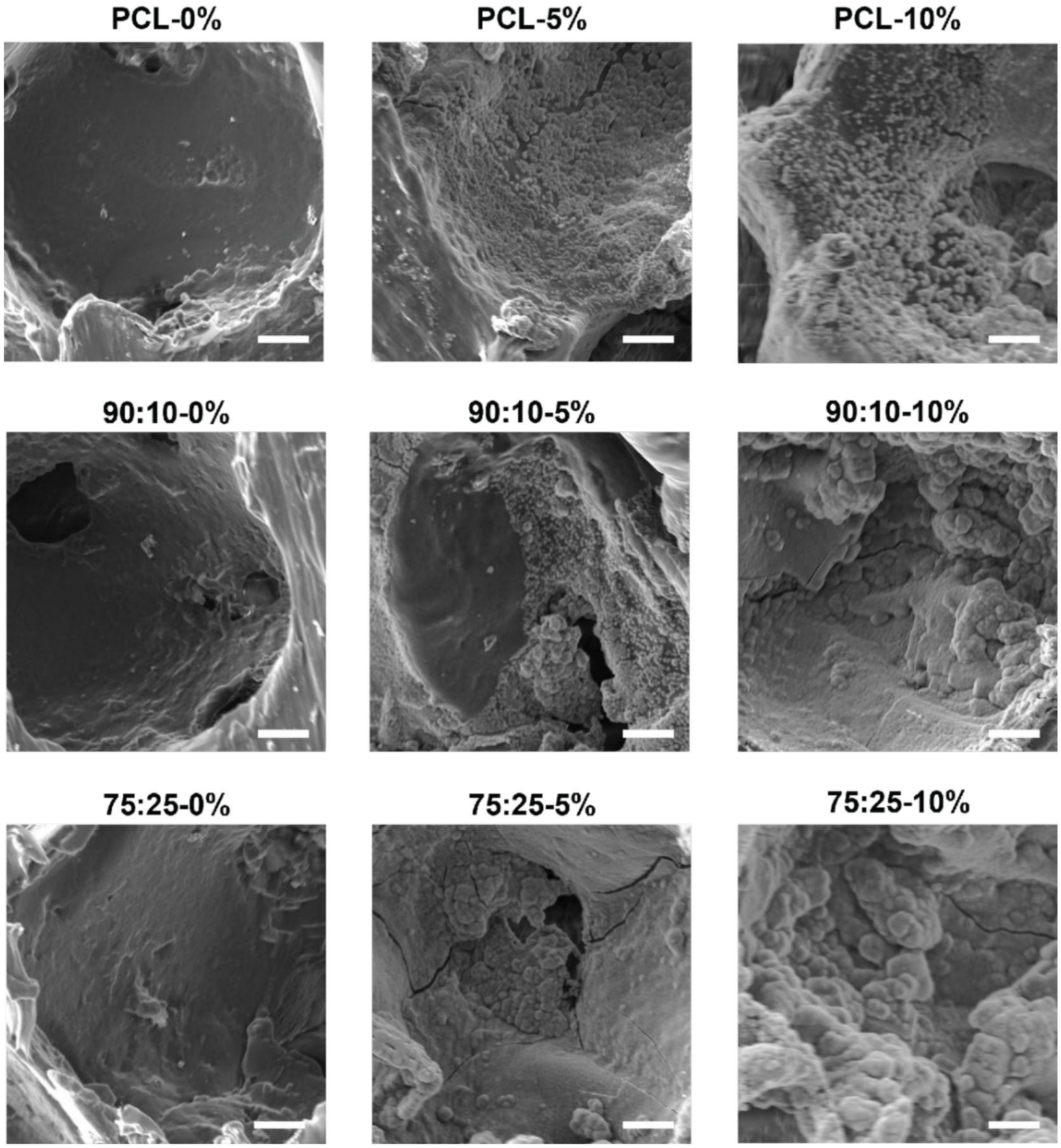

Figure S7. SEM images of scaffold cross sections after 2 weeks of 1X SBF exposure. Percentages refer to wt% of BG incorporated into each scaffold. Scale bars = 50 μm.

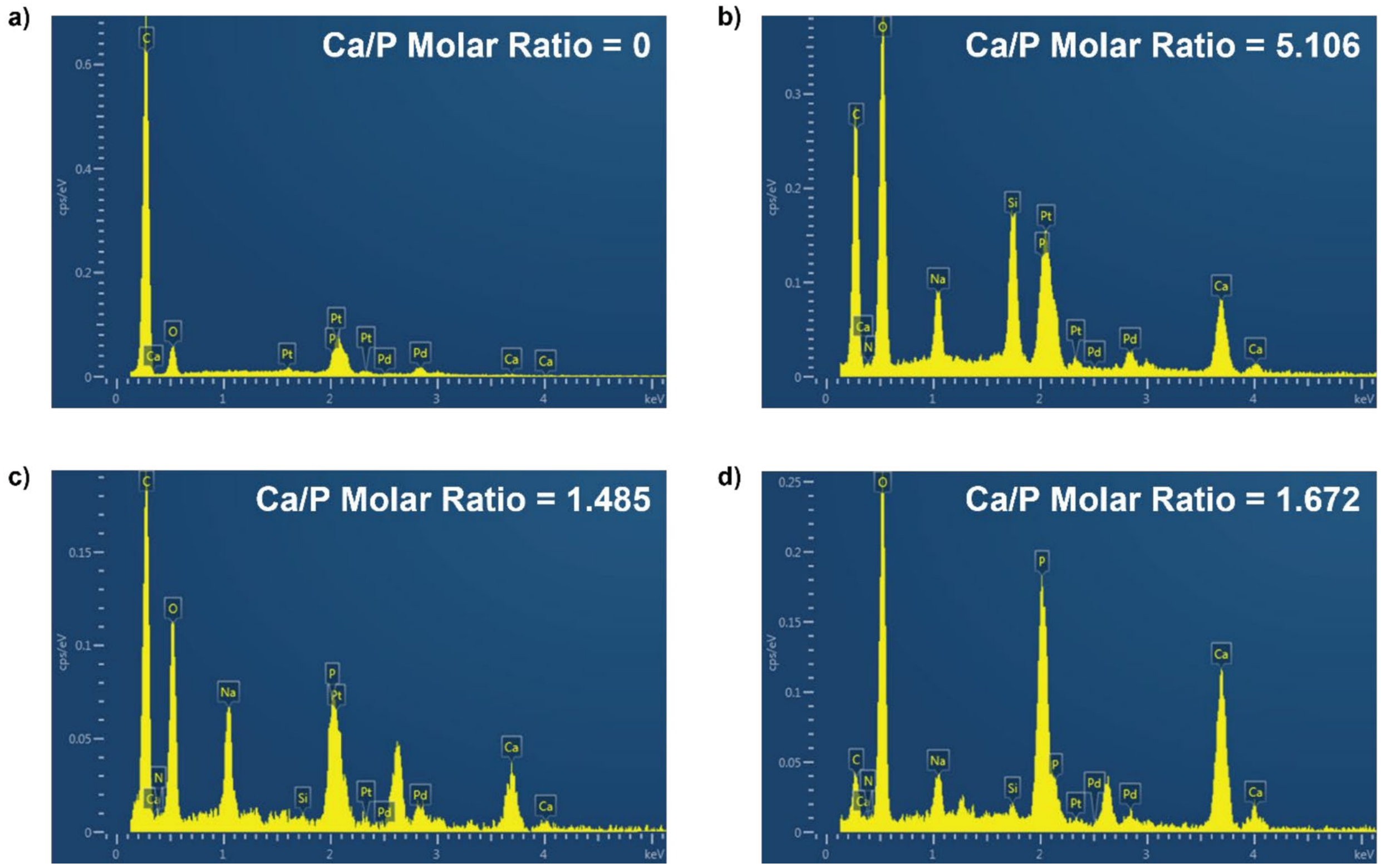

Figure S8. Representative EDS spectra of (a) 75:25-0% scaffold, (b) BG particle on scaffold, (c) 75:25-5% scaffold (t = 1 day, 1X SBF exposure), and (d) 75:25-5% scaffold (t = 2 weeks, 1X SBF exposure). Percentages refer to wt% of BG incorporated into each scaffold.

Table S8. Scaffold compressive mechanical properties prior to 1X SBF exposure. Percentages refer to wt% of BG incorporated into each scaffold. (Note: PCL data previously reported [2]; 90:10-0% and 75:25-0% data previously reported [1]).

Scaffold	Modulus (MPa)	Strength (MPa)	Toughness (MJ m^{-3})
PCL-0%	7.38 ± 1.87	31.85 ± 7.23	5.16 ± 0.89
PCL-5%	13.3 ± 2.72	50.38 ± 4.19	8.09 ± 0.68
PCL-10%	6.04 ± 2.31	39.19 ± 4.72	5.55 ± 0.89
90:10-0%	4.25 ± 1.39	29.45 ± 4.58	4.38 ± 0.88
90:10-5%	9.58 ± 2.37	42.78 ± 4.47	5.40 ± 1.80
90:10-10%	6.22 ± 3.01	26.56 ± 10.54	4.70 ± 1.66
75:25-0%	4.20 ± 0.51	25.97 ± 5.40	3.92 ± 0.73
75:25-5%	6.95 ± 3.28	34.33 ± 8.22	5.76 ± 1.56
75:25-10%	5.51 ± 1.13	31.28 ± 10.23	5.48 ± 1.67

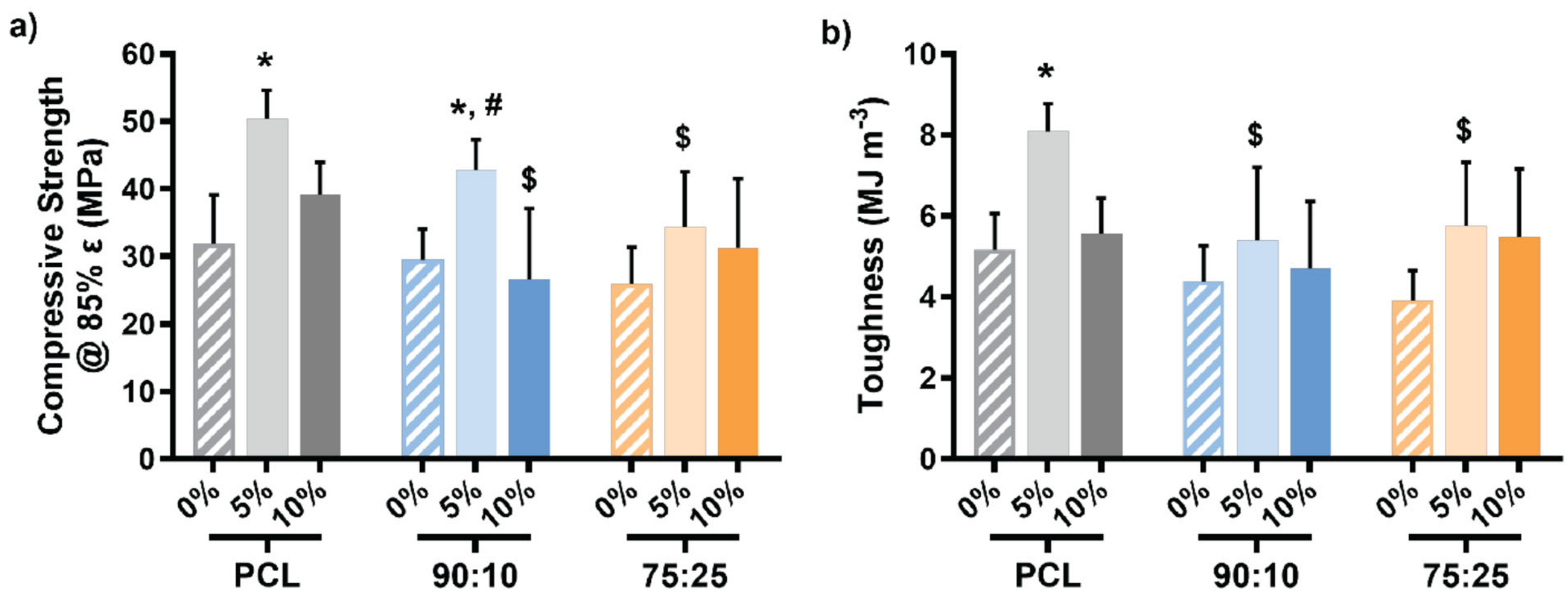

Figure S9. (a) Compressive strength and (b) compressive toughness of scaffolds. Percentages refer to wt% of BG incorporated into each scaffold. * $p < 0.05$ vs. analogous scaffold with 0% BG; $ p < 0.05 vs. PCL scaffolds with same wt% BG; # $p < 0.05$ vs. PCL-0% (control). (Note: PCL data previously reported [2]; 90:10-0% and 75:25-0% data previously reported [1]).

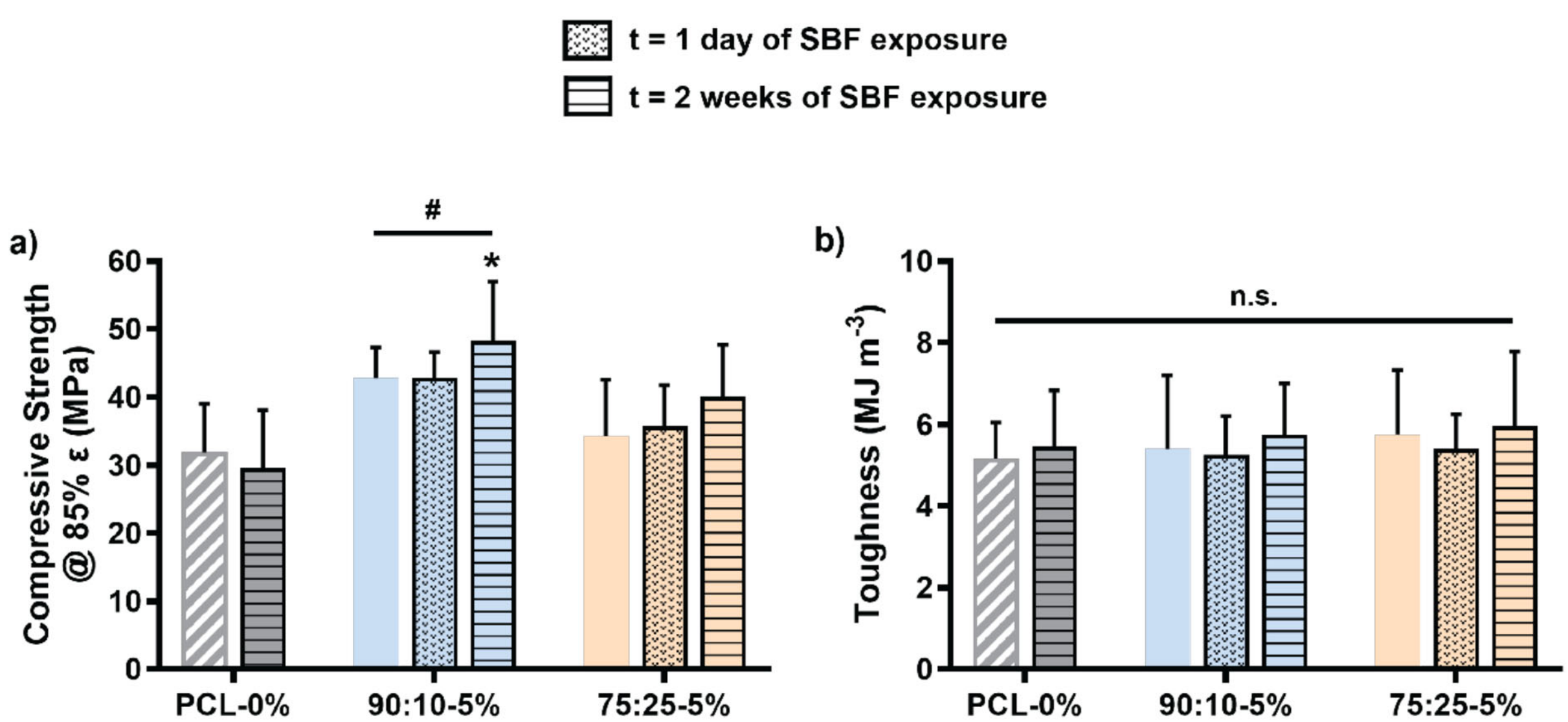

Figure S10. (a) Compressive strength and (b) compressive toughness of PCL-0%, 90:10-5%, and 75:25-5% scaffolds after exposure to 1X SBF. Percentages refer to wt% of BG incorporated into each scaffold. * $p < 0.05$ vs. analogous pre-mineralized scaffold; # $p < 0.05$ vs. pre-mineralized PCL-0% (control). (Note: pre-mineralized PCL-0% data previously reported [2]).

Table S9. Scaffold compressive mechanical properties after 1X SBF exposure. Percentages refer to wt% of BG incorporated into each scaffold. (Note: "—" indicates that PCL-0% was not tested after 1 day of 1X SBF exposure).

Scaffold	Modulus (MPa)		Strength (MPa)		Toughness (MJ m^{-3})	
	1 Day	2 Weeks	1 Day	2 Weeks	1 Day	2 Weeks
PCL-0%	—	7.10 ± 1.65	—	29.60 ± 8.54	—	5.42 ± 1.37
90:10-5%	9.80 ± 2.10	14.67 ± 1.15	42.90 ± 3.75	48.21 ± 8.74	5.26 ± 0.95	5.75 ± 1.25
75:25-5%	7.21 ± 1.65	12.42 ± 1.25	35.78 ± 5.92	40.05 ± 7.63	5.40 ± 0.85	5.96 ± 1.82

References

1. D. T. Dixon, A. G. Shields, S. J. Stafslien, L. Vander Wal, and M. A. Grunlan,"Hybrid Chitosan/PCL Shape Memory Scaffolds with Potential for Bone Regeneration and Infection Resistance," *ACS Biomaterials Science & Engineering* 11 (2025): 5627–5637, https://doi.org/10.1021/acsbiomaterials.5c01160.

2. B. M. Nitschke, E. A. Butchko, M. N. Wahby, K. M. Breining, A. E. Konz, and M. A. Grunlan,"Shape Memory Polymer Bioglass Composite Scaffolds Designed to Heal Complex Bone Defects," *ACS Biomaterials Science & Engineering* 10 (2024): 6509–6519, https://doi.org/10.1021/acsbiomaterials.4c01073.
